# Mechanism by which water and protein electrostatic interactions control proton transfer at the active site of channelrhodopsin

**DOI:** 10.1371/journal.pone.0201298

**Published:** 2018-08-07

**Authors:** Suliman Adam, Ana-Nicoleta Bondar

**Affiliations:** Freie Universität Berlin, Department of Physics, Theoretical Molecular Biophysics Group, Berlin, Germany; Universidade Nova de Lisboa Instituto de Tecnologia Quimica e Biologica, PORTUGAL

## Abstract

Channelrhodopsins are light-sensitive ion channels whose reaction cycles involve conformation-coupled transfer of protons. Understanding how channelrhodopsins work is important for applications in optogenetics, where light activation of these proteins triggers changes in the transmembrane potential across excitable membranes. A fundamental open question is how the protein environment ensures that unproductive proton transfer from the retinal Schiff base to the nearby carboxylate counterion is avoided in the resting state of the channel. To address this question, we performed combined quantum mechanical/molecular mechanical proton transfer calculations with explicit treatment of the surrounding lipid membrane. The free energy profiles computed for proton transfer to the counterion, either via a direct jump or mediated by a water molecule, demonstrate that, when retinal is all-*trans*, water and protein electrostatic interactions largely favour the protonated retinal Schiff base state. We identified a conserved lysine group as an essential structural element for the proton transfer energetics in channelrhodopsins.

## Introduction

Proton transfer reactions are of vital importance for biology [1,2]. Transfer of protons from donor to acceptor groups occurs, for example, during oxygenic photosynthesis [3] and during the reaction cycles of enzymes and membrane transporters that have been implicated in human disease [4,5]. A fundamental issue for proteins whose reaction cycles involve proton transfer reactions is that the timing of the proton transfer event must be tightly controlled. A proton transfer reaction is typically coupled to changes in the protein conformational dynamics and can couple to other chemical reactions such as breaking of peptide bonds. Channelrhodopsins (ChRs) are light-driven cation channels, responsible for phototaxis in green algae [6]. ChRs are used in modern neurobiological applications [7] and present an intriguing structural arrangement at their active site, where the primary proton donor—the retinal Schiff base—is found in a highly polar environment that includes two nearby carboxylate groups. Yet, proton transfer from the retinal Schiff base to one of these nearby carboxylate group occurs only once interactions at the active site have been destabilized upon photoisomerization of the retinal chromophore from all-*trans* to 13-*cis*, thus allowing ChRs to avoid wasteful deprotonation of all-*trans* retinal. Knowledge of the molecular mechanism by which ChRs stabilize the protonated state of the retinal Schiff base would be valuable for our general understanding of structural and energetic determinants of proton transfer in polar protein environments, and could assist with fine-tuning properties of ChRs for their usage as optogenetic tools.

ChRs are members of the large family of microbial rhodopsins [8], which are transmembrane proteins characterized by a seven-transmembrane helix fold, as first observed for bacteriorhodopsin [9], with the retinal molecule covalently bound via a protonated Schiff base to a lysine amino acid residue on helix G. Among the best-studied ChRs are channelrhodopsin-1 (ChR1) and channelrhodopsin-2 (ChR2) from *Chlamydomonas reinhardtii*. Various issues pertaining to the reaction cycles of these two proteins have been characterized using electrophysiological [6, 10, 11] and spectroscopic methods [12–20]. An electron microscopy structure of ChR2 has been solved at a low resolution of 6 Å [21], and a chimaera made of helices A–E of ChR1 and helices F–G of ChR2 (C1C2) has been solved at a resolution of 2.3 Å using X-ray crystallography [22]. Later, a blue-shifted C1C2 mutant was solved at 2.5 Å resolution [23], and most recently, the crystal structures of wild-type ChR2 from *C*. *reinhardtii* and its C128T mutant were solved at a resolution of 2.39 Å and 2.7 Å, respectively [24].

**Fig 1:**
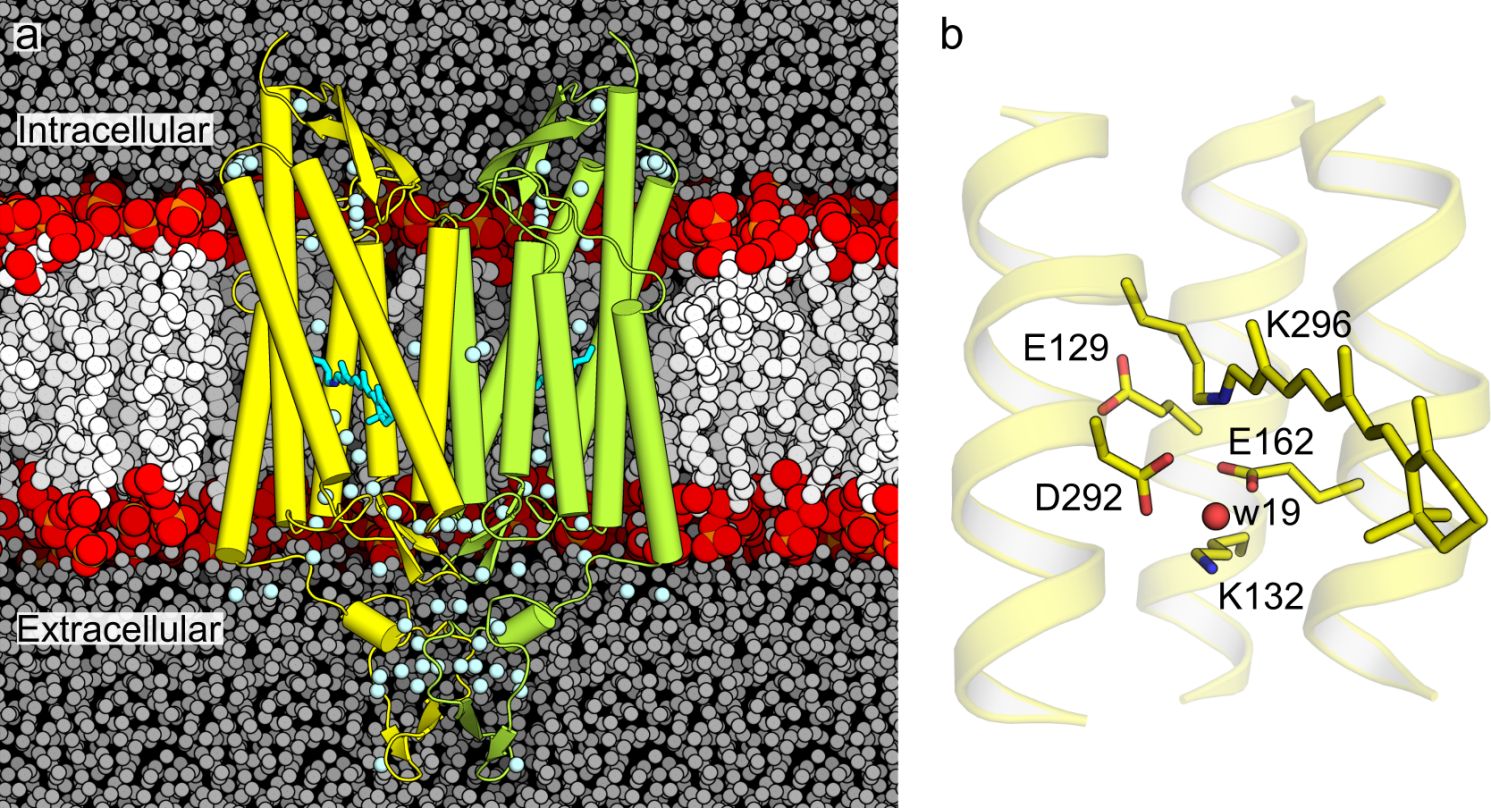
Structure and active site interactions of C1C2. (a) Cut-away view of the C1C2 dimer in a hydrated POPC lipid membrane environment. The protein coordinates are from the crystal structure of C1C2 (PDB ID: 3UG9 [22]). The C1C2 monomers are shown in yellow and lime cartoons, with water oxygen atoms from the crystal structure shown as small light blue spheres. The lipid membrane is shown with white spheres for all heavy atoms, except for phosphorus and oxygen atoms, which are shown in orange and red, respectively. (b) Close-up of the retinal Schiff base region from the crystal structure. The retinal molecule and selected protein side chains are shown in stick representation with carbon, oxygen and nitrogen atoms highlighted in yellow, red and blue, respectively. The active site water molecule w19 is shown as a red sphere.

The crystal structures of C1C2 [22] and of wild-type ChR2 [24] placed the nitrogen atom of the protonated Schiff base within hydrogen-bonding distance of the carboxylate oxygen atoms of the nearby carboxylate groups from helices C and G (E162 and D292 in C1C2, see Fig 1B). In both crystal structures [22, 24], the retinal Schiff base lacks a direct hydrogen bond to water. This structural arrangement at the active site of ChRs is intriguing, because it raises the fundamental question as to how ChRs avoid deprotonation of the retinal Schiff base in the all-*trans* resting state of the channel. In bacteriorhodopsin, for example, hydrogen-bonding to an active site water molecule (w402) is known to be essential for the stability of the protonated Schiff base state [25–29]. Computations for proton transfer from the retinal Schiff base to the carboxylate counterion in bacteriorhodopsin (D85) indicate that the absence of w402 significantly lowers the energetic penalty for retinal deprotonation [28, 29].

Lack of electron density for a water oxygen atom within hydrogen-bonding distance from the retinal Schiff base in the crystal structure of C1C2 [22] and ChR2 [24] might suggest that ChRs, unlike bacteriorhodopsin, may rely on structural elements other than hydrogen-bonding water to stabilize the proton on the Schiff base of all-*trans* retinal. Important indications that ChRs could use a different strategy for controlling the protonation state at their active sites are provided by experimental data on ChRs variants [30, 31]. In *C*. *augustae* ChR1, the all-*trans* retinal resting state of has E169 (corresponds to E162 in C1C2) protonated and D299 (corresponds to D292 in C1C2) negatively charged [32]. Deprotonation of the retinal Schiff base is thought to occur via a two-step mechanism, whereby a proton is first transferred from E169 to D299 and, in the second step, the Schiff base proton is transferred to D299 [32]. The observation that E169 is protonated in the resting state of *C*. *augustae* ChR1 [32] appears compatible with initial suggestions based on estimations of pK_*a*_ values, that E162 (Fig 1) is protonated in the crystal structure of all-*trans* C1C2 [22]. By contrast, FTIR spectroscopy data were interpreted to suggest that both E162 and D292 of C1C2 are negatively charged, and that E162 hydrogen-bonds directly with the retinal Schiff base [19]. In the case of *C*. *reinhardtii* ChR2, D253 (corresponds to D292 in C1C2) was identified as the primary proton acceptor of the retinal Schiff base proton [17].

An important structural element that distinguishes some of the ChR variants is whether they contain a specific lysine group on helix B (corresponding to K132 in C1C2) or not [31] (Fig 1B). K132 is present in *C*. *reinhardtii* ChR1 and ChR2, but not in *C*. *augustae* ChR1, where it is replaced by a phenylalanine group [31, 33]. Based on observations from site-directed mutagenesis, it has been proposed that K132 controls the effective charge of the counterions E162 and D292 [31]. Inside C1C2, the presence of complex hydrogen-bonding networks that involve K132 has been suggested by FTIR [19] and was observed in a recent molecular dynamics (MD) study [34].

The reaction mechanism for proton transfer is given by the sequence of structural changes and associated energetics along the pathway from the reactant state, where the proton is on the proton donor group and the proton acceptor group is negatively charged, to the neutral product state, where both the proton donor and acceptor groups are electrostatically neutral [35]. The reaction mechanism will then be given by the transition state of the pathway [29, 35]. In protein environments, where the proton donor and acceptor groups can engage in complex intramolecular interactions, more than one reaction pathway is possible [29, 36, 37]. In the case of the all-*trans* retinal resting state of ChRs, stability of the protonated retinal Schiff base would require a proton transfer energy profile characterized by unfavourable reaction energetics, i.e. the free energy of the product state would need to be sufficiently larger than that of the reactant state. By dissecting the reaction energetics of such proton transfer pathways, we can understand what the molecular interactions are that help control the protonation states at the active site of the protein.

To find out the structural and energetic determinants of the protonated retinal Schiff base state in ChRs we pursued systematic computations of proton transfers in C1C2. We used a combined quantum mechanical/molecular mechanical (QM/MM) approach [38, 39] that allows us to treat with quantum mechanics (QM) the breaking and forming of covalent bonds during proton transfer and to account for the protein environment with a simpler, classical mechanical (MM) description. Because protein flexibility [40] and water interactions [36] can impact significantly the energetics of proton transfer pathways, we performed computations of the potential of mean force (PMF) for proton transfer at 300 K, in the flexible environment of C1C2 embedded in a hydrated lipid membrane. The results of our computations indicate that K132 and active site water molecules largely stabilize the protonated Schiff state in all-*trans* retinal C1C2.

## Methods

We performed MM and QM/MM all-atom MD simulations of ChR using the CHARMM36 protein [41, 42] and lipid [43] force fields, the ion parameters of Roux and coworkers [44] and the TIP3P water model [45]. The retinal parameters were based on work performed by Hayashi [27, 46], Nina [47], Baudry [48] and Tajkhorshid [49–51]. As QM method, we used the self-consistent charge density functional tight binding (SCC-DFTB) method [52], whose applicability to describing retinal geometry and proton transfer energetics has been documented extensively [29, 53–55].

### MM simulations of all-*trans* C1C2

As starting coordinates, we used the crystal structure of the chimaera C1C2 (PDB ID: 3UG9 [22]). Using the Phyre2 web portal [56], we modelled amino acid residues 110–117 that were missing from the crystal structure of C1C2. We assembled the protein dimer with PISA [57] and linked the dimer with three disulphide bridges for C66, C73 and C75. We used standard protonation for all amino acid residues, except for E122, E129 and D195, which were considered protonated based on experimental information for dark-state channelrhodopsin [13, 16, 17, 20, 58–60], and E162, for which both neutral and negatively charged states were generated in independent simulations. Hydrogen atoms were constructed with HBUILD in CHARMM [61]. The protein dimer was placed inside a hydrated 1-palmitoyl-2-oleoyl-sn-glycero-3-phosphocholine (POPC) lipid membrane using the CHARMM-GUI [62, 63] Membrane Builder [64–66]. POPC lipid membranes are often employed to study the dynamics of membrane proteins, including ChRs [34, 67–72]. The complete simulation consisted of the C1C2 dimer, 86 water molecules found in the crystal structure (43 per monomer), 300 POPC molecules and ~28 500 bulk water molecules in a simulation box of size 110×110×120 Å^3^. For charge neutrality, we added two chloride ions to setups with protonated E162.

The MM simulations were performed using NAMD [73, 74] with a Langevin dynamics scheme and a Nosé–Hoover Langevin piston [75, 76] at a temperature of 300 K. We constrained covalent bonds involving hydrogen atoms using the SHAKE algorithm [77].

The first nanosecond of heating and equilibration was done following the CHARMM-GUI protocol [62, 63], while maintaining an integration time step of 1 fs and reassigning velocities every 0.5 ps. This was followed by two additional steps of equilibration of 2 ns each, where we placed on the protein and lipid heavy atoms a harmonic constraint of 5 kcal/mol/Å^2^. Starting from the last 2 ns of the equilibration, we used to the reversible multiple time step integration scheme [78, 79] with steps of 1 fs for the bonded forces, 2 fs for short-range non-bonded and 4 fs for long-range non-bonded interactions. With the exception of the first 100 ps, where a canonical ensemble (*NVT*) was used, we used an isothermal–isobaric ensemble (*NPT*) with isotropic cell fluctuations at a pressure of 1 bar. After 5 ns of equilibration, production runs were started. The Langvin damping coefficient was 1 ps^-1^ during equilibration and 5 ps^-1^ during production runs. We generated 250 ns trajectories for each simulation of all-*trans* C1C2. We saved coordinates every 10 ps.

### Modelling of the K132A mutant

To understand the impact K132 has on the proton transfer energetics of the retinal Schiff base, we performed additional calculations with the K132 to alanine (K132A) mutant of C1C2. K132A has faster kinetics than wild-type C1C2 and changes the ion selectivity of the channel from an unselective one to a potassium channel [22].

For the mutant computations, we used the wild type starting coordinates of C1C2 with protonated and unprotonated E162. We performed the equilibration as described above for wild-type C1C2, with the only exception being that the 5 kcal/mol/Å^2^ harmonic constraint placed on the protein atoms was not relaxed. The equilibration was followed by 20 ns of simulation with the protein still constrained, after which we used CHARMM to mutate K132 to alanine in both monomers of C1C2. We then repeated the equilibration procedure using the same approach as described above for the wild type simulations, before prolonging the trajectories of the production runs to 110 ns each.

### QM/MM computations of all-*trans* C1C2

We employed the CHARMM software package [42, 61, 80] with the SCCDFTB module [81] to perform the QM/MM calculations using third-order SCC-DFTB [52, 82, 83]. To prepare the all-*trans* QM/MM systems, we took one snapshot each from the end of each of the MM trajectories. In case of the wild type simulations, we saved the coordinates at the end of the 250 ns production run. We used the same protocol for the QM/MM simulations of the K132A mutant, which were started from the end of their MM production run at 110 ns. During the first 50 ps of QM/MM dynamics, we placed on all heavy atoms a mass-scaled harmonic positional constraint given by the constraining potential:

$$U(x)=p\sum_{i}\left\{k_{i}m_{i}{\left(x_{i}-x_{i,ref}\right)}^{2}\right\},$$

where, for an atom *i*, *k*_*i*_ is the force constant of the constraint in kcal/mol/Å^2^, *m*_*i*_ is the atomic weight, *x*_*i*_ is the current position in Å, *x*_*i*,*ref*_ is the position of a reference set of coordinates in Å, and *p* is a pre-factor used to scale the constraint. We started with a pre-factor value of 1.0, and every 10 ps we reduced the value by 0.25 until 0 was reached. The equilibration was followed by a production run of 1 ns.

Because on the 1 ns timescale water molecules inside the Schiff base region could be replaced by water molecules initially located farther away from the Schiff base, we constrained MM water molecules at the entrance of the Schiff base region by 2.5 kcal/mol/Å^2^. This keeps QM water molecules inside the Schiff base region without having to directly apply a constraining potential to them. A similar approach has been used before [69].

The QM/MM dynamics calculations were run using the Leapfrog Verlet algorithm and a canonical ensemble with a damping constant of 5 ps^-1^ at a temperature of 300 K. We used an integration time step of 1 fs and saved coordinates every 1 ps.

### Choice of QM region

Because ChR is a dimer, the QM/MM treatment could be applied to either one of the active sites in the two C1C2 monomers. For simplicity, we treated the active site of only one of the protein monomers with QM, and we performed independent QM/MM MD and proton transfer computations in which the QM treatment was applied either to monomer 1 or to monomer 2 of C1C2.

The QM region includes protein and water groups that could directly participate in the proton transfer reaction as well as selected groups from the immediate vicinity of the proton donor and acceptor groups. We thus treated with QM the whole retinal molecule and the K296 side chain, the retinal is covalently bound to, the side chains of E162 and D292, which we consider as potential proton acceptor groups, and the side chains of the charged groups E129 and K132, which may sample direct hydrogen bonds with E162 and D292 (Fig 1B). Three water molecules that were located close to the Schiff base nitrogen in the starting crystal structure, were also included in the QM region. Later in the text, these three water molecules are labelled as w1, w2 and w3. A similar QM region that did not include E129 has been used in previous computations on a homology model of ChR2 [67]. [84]

We placed link atoms on CB for D292, on CD for K132 and on CG for E129, E162 and K296. Studies of a lysine–retinal model system in vacuo revealed only a negligible impact on proton transfer energetics caused by our choice link atom placement. Previous test computations had shown that using the CB-CG bond as frontier bond for K296 does not interfere with the geometry or deprotonation energy of the retinal [54]. A link atom on the CB of an aspartate side chain allows for the entire side chain to be treated with QM and has been used before [54]. We used the divided frontier link atom scheme to ensure charge neutrality at the interface, while at the same time preserving the charge of the backbone N–H group of D292, where the QM region was cut next to the backbone [84]. In K132A, we treated residue 132 with MM. The QM/MM computations were initiated from the end of each MM trajectory. Depending on the simulation system—wild-type or K132A, with or without QM water—there were 82 to 103 atoms in the QM region.

### Proton transfer calculations

Proton transfer calculations were performed starting from the last coordinate snapshot of the QM/MM simulations using Alan Grossfield’s implementation [85] of the Weighted Histogram Approach Method (WHAM) [86], an extension of the umbrella sampling method [87, 88]. By using WHAM, the PMF of a reaction coordinate can be derived combining bins with different constraints placed on the reaction coordinate.

The reaction coordinate *D* of the proton transfer was defined as the difference between donor–donor hydrogen distance and acceptor–donor hydrogen distance:

$$D=d_{DH}-d_{AH},$$

where *d*_*DH*_ is the distance between donor and donor hydrogen, and *d*_*AH*_ is the distance between donor hydrogen and acceptor. We used the restrained distances (RESD) command [89] inside CHARMM to constrain *D* to generate bins for sampling the deprotonation potential of mean force. The constraint is given by:

$$E=\frac{1}{2}k{\left(D-D_{ref}\right)}^{2},$$

where *k* is the constraining force constant in kcal/mol/Å^2^, and *D*_*ref*_ is the minimum of the constraint in Å. We used a force constant *k* of 150 kcal/mol/Å^2^.

To generate starting points to begin sampling the bins for the WHAM analysis, we performed a short initial sampling as follows: Using the initial coordinates and starting from a value of *D*_*ref*,*0*_ = −1.2 Å for an amino acid as acceptors, or *D*_*ref*,*0*_ = −1.0 Å for a water molecule as acceptor, the RESD constraint was applied; the system was equilibrated for 2.5 ps and a restart file was saved; *D*_*ref*_ was decreased by 0.1 Å and another equilibration followed. This was done until *D*_*ref*_ = −1.7 Å was reached. A second run was started from the initial coordinates; this time *D*_*ref*,*0*_ was increased by 0.1 Å, followed by a 2.5 ps equilibration, until *D*_*ref*_ = 1.7 Å was reached. Once all 35 restart files had been generated, a 100 ps equilibration was started for each bin, using the respective *D*_*ref*_ value. The last 50 ps of each bin were employed for the WHAM calculations, with the values of the reaction coordinate *D* being saved at every time step.

To characterize structural and energetic determinants of the proton transfer energetics, we performed additional WHAM analyses with reduced systems as summarized below.

In the first test system, denoted as the PROT setup, we started from the end of the QM/MM simulation of C1C2 and deleted all lipid molecules and all water molecules farther than 5 Å away from the protein. The PMF computation on the PROT setup was then performed without additional geometry optimization. We performed 10 ps of equilibration, and then performed PMF computations using the same protocol as described above for the complete system.

In the second test system, denoted as DRY, starting from the end of the QM/MM simulation of C1C2 as well, we deleted, except for the water molecule closest to the Schiff base nitrogen, all water molecules within 4 Å of the Schiff base nitrogen or the carboxyl(ate) oxygen atoms of E162 or D292. In the DRIER setup, we deleted all water molecules within 4 Å of the Schiff base nitrogen or carboxyl(ate) oxygens without any exception. We found that the deletion of QM waters in the DRY test setup led to the need for a brief energy minimization before the initial 10 ps equilibration and the PMF computation could be performed.

Finally, the crystal setup consisted of the starting crystal structure of a C1C2 monomer (PDB ID: 3UG9 [22]). In the crystal structure, there is only one water molecule (w19) in the Schiff base region; the distance between the oxygen atom of w19 and the Schiff base nitrogen of 4.43 Å, is too long for a direct hydrogen bond. Consequently, the PMF computations on the crystal test system considered only proton transfer pathways from the retinal Schiff base to E162 and D292.

To further test the importance of hydration for the energetics of the proton transfer pathways, we used the DOWSER plugin [90, 91] inside VMD [92] to generate possible missing water molecules in the Schiff base region, and then performed PMF computations. These test systems are labelled here as the DOWSER setups.

To preserve the shape of the protein during computations on the PROT, crystal and DOWSER test systems described above, we constrained coordinates of the heavy atoms of the loop regions and of all water molecules within 5 Å of the loop regions by using a harmonic constraint of 10 kcal/mol/Å^2^ and 5 kcal/mol/Å^2^ for loops and water molecules, respectively.

All molecular graphics were prepared using PyMOL [93].

Unless specified otherwise, all average values were computed from the last 50 ns of each MM simulation.

## Results and discussion

We performed MM and QM/MM studies on the C1C2 chimaera of *C*. *reinhardii* ChR (PDB ID: 3UG9 [22]) to assess the impact the environment of the Schiff base has on the stability of the Schiff base proton in the dark state.

A summary of all MM simulations performed is given in Table 1. For each simulation and its repeat (indicated by a prime symbol), the C_α_ root-mean-square deviation (RMSD) had achieved plateau values for the last 50 ns that we used here for data analysis (S1 Fig). We note that during the last 50 ns of the wild type simulation with unprotonated E162 the RMSD for the entire protein was 2.6 ± 0.1 Å, and that the RMSD profile had reached its plateau within less than 10 ns (S1 Fig). The α-helical regions stayed even closer to the crystal structure with an RMSD of 1.1 ± 0.1 Å. This observation of a very low α-helical RMSD was shared across all simulations, with average RMSDs of 1.1–1.5 Å. Average RMSD values for the whole protein were between 2.3–3.1 Å, indicating overall good structural stability. For the loop regions, we obtained larger RMSD values of 3.3–4.4 Å; such values are consistent with previous indications from MD simulations that the loop regions can be the most dynamic part of a protein, such that their motions cannot be sampled sufficiently on the timescale of ∼100 ns [94].

**Table 1:** List of MM simulations performed for wild-type C1C2 and for the K132A mutant.

Simulation	Protein	E162	Length
simWu	wild type	unprotonated	250 ns
simWu′	wild type	unprotonated	250 ns
simWp	wild type	protonated	250 ns
simWp′	wild type	protonated	250 ns
simMu	K132A	unprotonated	110 ns
simMu′	K132A	unprotonated	110 ns
simMp	K132A	protonated	110 ns
simMp′	K132A	protonated	110 ns

A prime symbol indicates repeat simulations. See S1 Fig for C_α_ RMSD profiles of the simulations.

In what follows, we first summarize observations from the MM simulations, with focus on internal water molecules and hydrogen bonding at the retinal Schiff base region, before proceeding to discuss the QM/MM computations.

### Water molecules visit the intrahelical region of wild-type C1C2

**Fig 2:**
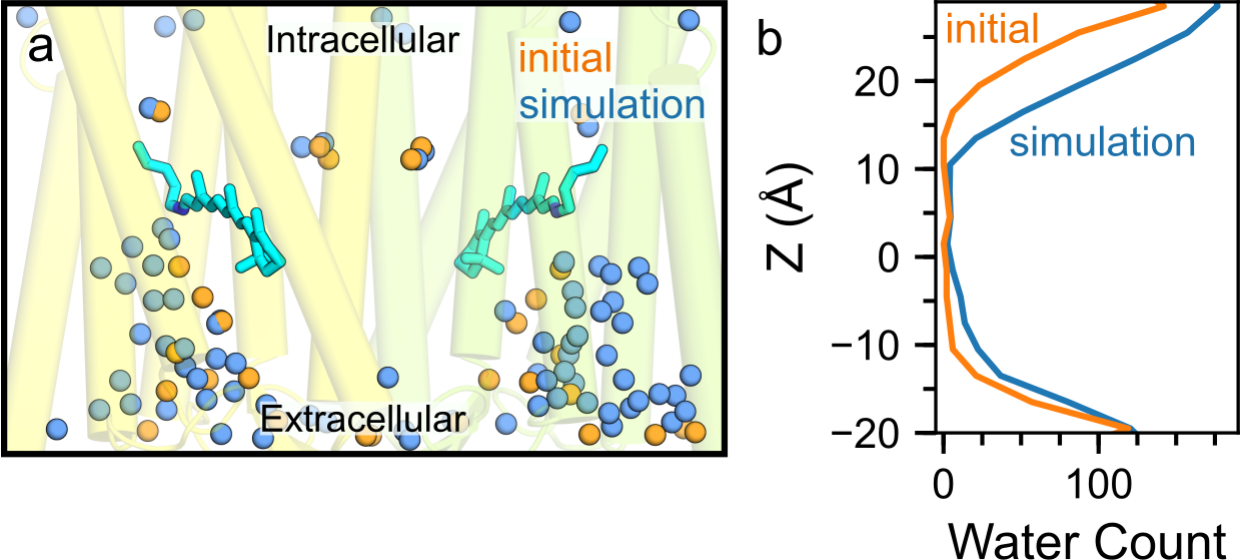
Hydration of the intrahelical region in wild-type C1C2 with unprotonated E162. (a) The number of water molecules along the membrane normal (z-axis) for water molecules within 6 Å of C1C2. The initial profile (orange) indicates the distribution of water molecules at the start of the simulations, that is, positions of waters inside C1C2 are as found in the starting crystal structure (PDB ID: 3UG9 [22]). The simulation profile (blue) indicates the average number of water molecules computed for the last 50 ns of simWu (wild-type C1C2 with unprotonated E162). Note the increase in the number of water molecules inside C1C2, in particular in the extracellular half. (b) Close view of C1C2, overlaying starting crystal water oxygen atoms (orange spheres) with water oxygen atoms taken from a coordinate snapshot of simWu (blue).

**Fig 3:**
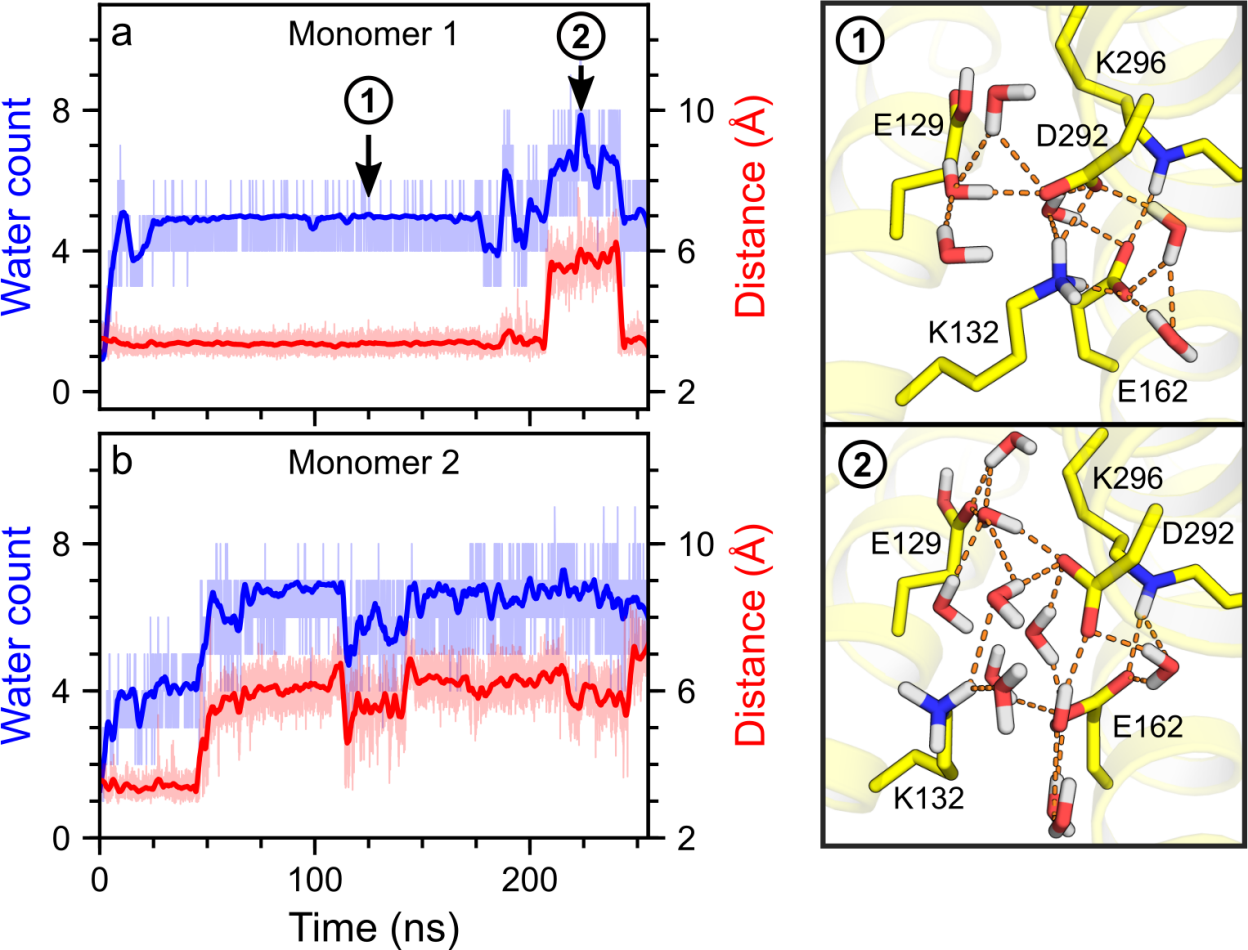
Dynamics of the K132–D292 interaction compared to the hydration of the active site. (a–b) Time series of the number of active site water molecules (blue profiles) and of the distance between NZ of K132 and CG of D292 (red profiles) in monomer 1 (a) and monomer 2 (b) of simWu. For each profile, thin lines indicate the calculated values, while thick lines indicate a smoothed fit. As water count we defined the number of water molecules forming hydrogen bonds with the Schiff base nitrogen or the carboxyl(ate) oxygens of E162/D292. The Pearson correlation coefficient for the K132–D292 distance and the water count was 0.63 and 0.79 for monomer 1 and monomer 2, respectively. Illustrations (1) and (2) are coordinate snapshots of monomer 1 with short (Illustration 1) and long (Illustration 2) distances between K132 and D292, as observed in the trajectory used for panel a. Note that there are more active site water molecules in Illustration 2 than in Illustration 1. Additional data analyses on the water count and the distance between E162 and D292 are given in S2 Fig.

The number and location of water molecules close to the proton transfer groups can significantly impact the proton transfer pathways and their associated energetics [29, 36]. Our simulations on C1C2 indicate that, when the protein is found in a flexible, hydrated lipid membrane environment, numerous water molecules visit the interhelical region of C1C2, where they sample hydrogen bonds with protein groups. Indeed, the number of water molecules inside the interhelical region of C1C2 was significantly higher in our simulations as compared to the starting crystal structure (Fig 2). The increase in the number of water molecules was more pronounced in the extracellular half of the protein, where water molecules entered rapidly close to the Schiff base region (Figs 2 and 3). An important outcome of waters entering the extracellular half of C1C2 was that, in all simulations performed on wild-type C1C2, there were on average ~5–6 water molecules hydrogen-bonding to either the Schiff base or E162/D292 (Figs 2 and 3, Table 2). In simulations on wild-type C1C2, regardless of the protonation state of E162, we observed the retinal Schiff base in both C1C2 monomers sampling direct hydrogen-bonding to water (Table 3).

**Table 2:** Number of water molecules forming hydrogen bonds with the schiff base nitrogen or the side chains of E162 or D292.

Simulation	Number of Water Molecules	
	Monomer 1	Monomer 2
*Wild-type C1C2*		
simWu	6.0 ± 1.0	6.6 ± 0.6
simWu′	4.4 ± 0.9	6.6 ± 0.9
simWp	5.6 ± 0.7	4.5 ± 0.7
simWp′	4.7 ± 0.8	4.2 ± 0.9
*K132A mutant*		
simMu	5.5 ± 0.7	4.7 ± 0.5
simMu′	7.5 ± 1.4	6.1 ± 0.6
simMp	5.3 ± 1.0	5.7 ± 0.7
simMp′	5.2 ± 0.9	5.4 ± 1.0

A prime symbol indicates repeat simulations.

**Table 3:** Hydrogen-bonding partners of the schiff base nitrogen during the last 50 ns of the MM simulations.

Sim	Monomer 1 (%)			Monomer 2 (%)		
	Water	E162	D292	Water	E162	D292
*Wild-type C1C2*						
simWu	19	92	—	68	—	51
simWu′	56	38	9	85	—	36
simWp	99	—	—	57	—	68
simWp′	60	—	54	96	—	—
*K132A mutant*						
simMu	82	—	38	—	—	100
simMu′	64	28	22	95	—	10
simMp	37	—	85	86	—	24
simMp′	54	—	66	61	—	60

For clarity, only percentages >3% are shown. A prime symbol indicates repeat simulations. Additional hydrogen-bonding data are summarized in S1–S4 Tables.

We note that, although the two C1C2 monomers had overall similar numbers of water molecules close to the retinal Schiff base region, for some simulations, the two monomers had slightly different levels of internal hydration (Table 2) and showed different tendencies for water hydrogen-bonding directly to the retinal Schiff base (Table 3). In SimWu′, for example, there were 4.4 ± 0.9 and 6.6 ± 0.9 water molecules in monomer 1 and 2, respectively, and direct hydrogen bonding between the retinal Schiff base and water was sampled only transiently (19%) in monomer 1, whereas in monomer 2 it was sampled frequently (68%) (Table 3). These differences in the occupancies of dynamic hydrogen bonds in the two C1C2 monomers could be interpreted to suggest that full sampling of the dynamics of some of the intramolecular interactions would require simulations to be prolonged to timescales beyond the 250 ns reported here (Table 2). To account for water active site interactions as suggested by the analyses of both C1C2 monomers, the QM/MM computations for proton transfers reported below were performed separately for monomer 1 and monomer 2.

### Hydrogen-bonding networks in the Schiff base region

Direct hydrogen bonding of water molecules with proton donor and/or acceptor groups can impact significantly the proton affinity and the geometry of these groups, and consequently, the proton transfer energetics [55, 95]. We found that, for both wild-type C1C2 and K132A the Schiff base could hydrogen-bond only to E162, D292 or with nearby water molecules (Tables 3 and 4). This observation is compatible with the C1C2 crystal structure (PDB ID: 3UG9 [22]), with suggestions from experiments that D292 is the primary proton acceptor [17, 22], that E162 could hydrogen-bond with the Schiff base [19], and with previous MD work on homology models of ChR [34, 67, 69, 70]. Importantly, however, details of water hydrogen-bonding at the active site depend significantly on the protonation state of E162.

**Table 4:** Hydrogen-bonding partners of the Schiff base nitrogen during the last 750 ps of the QM/MM simulations of C1C2.

Sim	Monomer 1 (%)			Monomer 2 (%)		
	Water	E162	D292	Water	E162	D292
*Wild-type C1C2*						
simWu	90	41	—	84	—	83
simWp	93	—	15	97	—	4
*K132A mutant*						
simMu	93	—	14	—	—	95
simMp	5	30	91	97	—	12

For clarity, only percentages >3% are shown.

**Fig 4:**
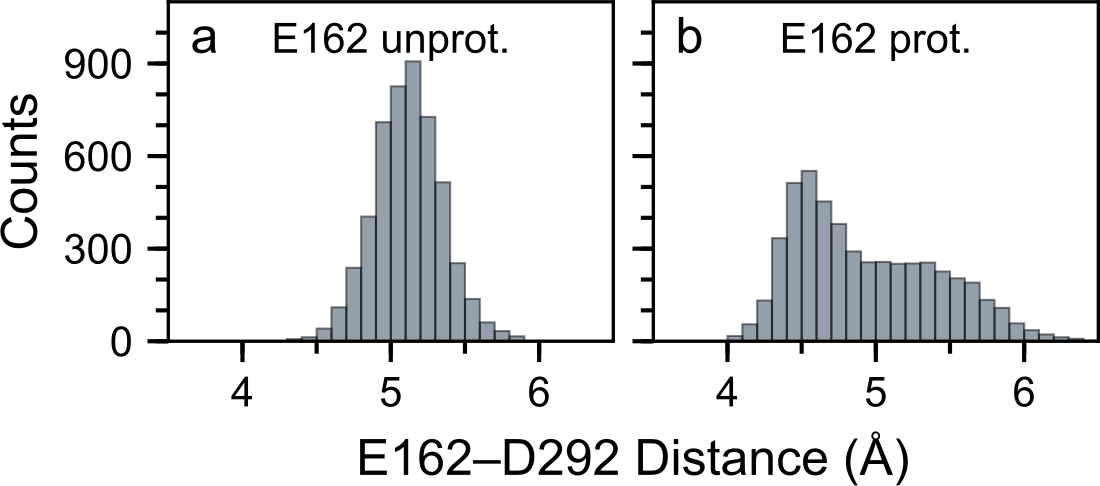
Dependence of the dynamics of the interaction between E162 and D292 on the protonation state of E162. We computed histograms of the distance between CD of E162 and CG of D292 in monomer 1 of simWu (panel a, wild-type C1C2 with unprotonated E162) and simWu′ (panel b, wild-type C1C2 with protonated E162). (a) In simWu, a one-water bridge mediates interactions between E162 and D292, and the distance between E162 and D292 is 5.1 ± 0.2 Å during the last 50 ns. (b) In simWp, D292 and the protonated E162 can either hydrogen-bond directly, resulting in the peak at ~4.5 Å, or interact via a one-water bridge, resulting in the shoulder from ~4.9 Å to ~5.4 Å. Additional data on the distance between E162 and D292 in simulations performed here are summarized in S3 Fig.

The distance between CD of E162 and CG of D292 informs on whether the carboxylate groups hydrogen-bond directly with each other, or via one or more water molecules (Fig 4). Indeed, the average E162–D292 distance was 5.2 Å when E162 is negatively charged (Fig 4A), which corresponds to a water-mediated interaction between the two carboxylate groups. In the case of protonated E162, we observed that the distribution of the distances between E162 and D292 had a peak at 4.6 Å, corresponding to a direct hydrogen bond between E162 and D292, and a shoulder at larger values of the distance, which corresponds to water-mediated bridging of the two carboxylate groups (Fig 4B); for comparison, the distance between E162 and D292 in the starting crystal structure is 4.78 Å [22].

The protonation state of E162 additionally had an impact on the dynamics of its interactions with the K132 side chain (Fig 3). For negatively charged E162, K132 hydrogen-bonded with either E162 or D292, or with both residues at the same time (S4 Fig). These dynamics of the interactions between K132 and the carboxylate counterions were associated with changes in the number of water molecules close to the retinal Schiff base region (Fig 3). In simulations with E162 negatively charged, an increase in the distance between K132 and D292 led to an increase of the number of water molecules in the active site and vice versa.

To further assess the relationship between the K132–D292 distance and active site hydration, we computed the linear correlation between the distance that characterizes the K132–D292 interaction and the number of active site water molecules within hydrogen-bonding distance of either the Schiff base or E162/D292. We found that the K132–D292 distance and the number of active site waters were strongly linearly correlated in simulations with unprotonated E162, with a linear correlation of ∼70%. The high correlation persisted even in the absence of direct hydrogen bonding between K132 and D292. Protonation of E162 eliminated this correlation and was associated with the K132–E162 salt bridge being broken. Taken together, these analyses suggest that K132 helps to control the access of waters to the active site of C1C2.

### K132A alters internal water dynamics and protein hydrogen bonding

K132, which is present in many of the ChR sequences [33], appears to be involved in important electrostatic interactions at the active site of ChR. K132 influences the effective charge of E162 and D292 [31] and affects the absorption maximum of the retinal [96]. These observations are compatible with our computations above that indicated that the dynamics of the K132–D292 distance correlates with the dynamics of water at the active site (Fig 3).

To further probe the role of K132 in helping control the hydrogen-bond dynamics at the active site of C1C2, we used the crystal structure of C1C2 [22] to model the K132A mutant, and we utilized MM simulations to evaluate the impact that the mutation would have on hydrogen bonding at the active site of C1C2. We found that, when the K132 side chain was absent, R159 on helix C, which initially faced the extracellular side, could orient itself towards the active site region and engage in largely stable interactions with E162 and D292, i.e. we could associate the K132A mutation with altered dynamics of interhelical hydrogen bonds in the extracellular half of C1C2, including the counterions E162 and D292 (S4 Table).

### Proton transfer pathways in all-*trans* C1C2

The MM simulations summarized above indicate that the active site of C1C2 is characterized by the presence of a dynamic hydrogen-bonding network that includes the retinal Schiff base, protein side chains and water molecules. The retinal Schiff base can interact with a water molecule, with E162 or with D292, its preferred interaction partner depending to some extent on the protonation state of E162 (Table 3). For unprotonated E162, we observed persistent hydrogen bonding between E162 and the retinal Schiff base for monomer 1 of C1C2, which was associated with lower occupancies of water hydrogen bonding of the retinal Schiff base in both simWu and the repeat simWu′ (Table 3).

**Fig 5:**
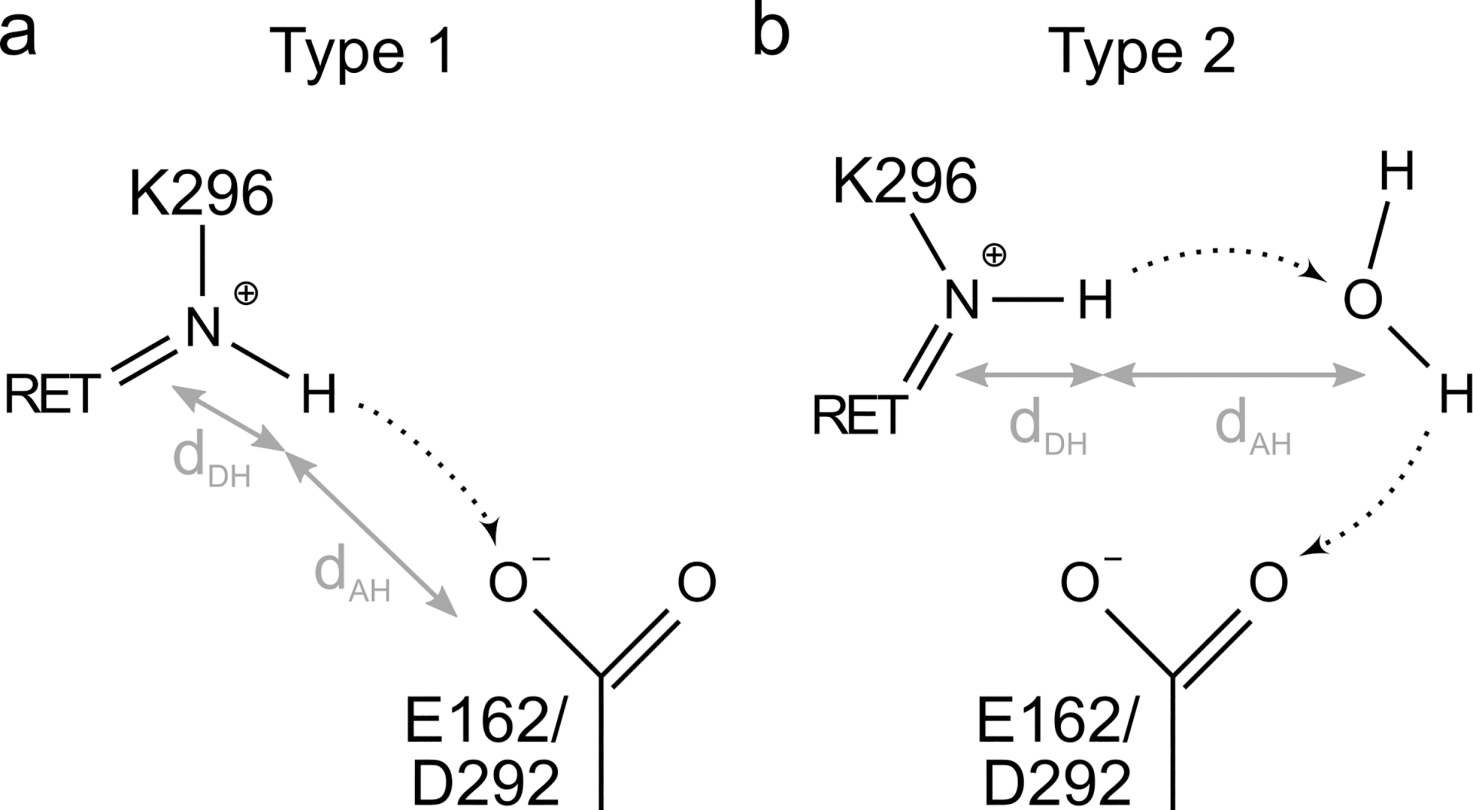
Illustrations of proton transfer. Proton transfer from the all-*trans* retinal Schiff base can occur via a direct jump or via an intermediate water molecule. The reaction coordinate *D* used for the proton transfer calculations is the difference between donor–hydrogen distance *d*_DH_ and acceptor–hydrogen distance *d*_AH_. (a) In a Type 1 proton transfer pathway, the proton directly moves from the retinal Schiff base to either E162 or D292. (b) In a Type 2 proton transfer pathway, the Schiff base proton is transferred to the carboxylate acceptor group via an intermediate water molecule.

To characterize the energetics of proton transfer in all-*trans* C1C2, we pursued an exhaustive set of QM/MM computations, in which we studied the dynamics and calculated proton transfer pathways for conformations of wild-type C1C2 that were representative for the active site dynamics observed. To this effect, we performed PMF computations for proton transfer from the retinal Schiff base to E162 or to D292, either directly (Type 1, Fig 5A) or via one water molecule that acted as intermediate carrier for the proton (Type 2, Fig 5B).

As starting point of our QM/MM proton transfer computations, we first used the last coordinate snapshot of the MM simulations simWu and simWp (Table 1) to perform 1 ns of QM/MM equilibration for each system. The occupancies of selected active site hydrogen bonds computed for these QM/MM simulations were mostly higher than in the corresponding MM simulations (Tables 3 and 4). We suggest that these differences in the occupancies of selected hydrogen bonds in MM vs QM/MM simulations are likely due to the fact that the QM/MM simulations, whose length is limited by the computational costs, provide an incomplete picture of the dynamics of the complex hydrogen-bonding network of C1C2.

**Fig 6:**
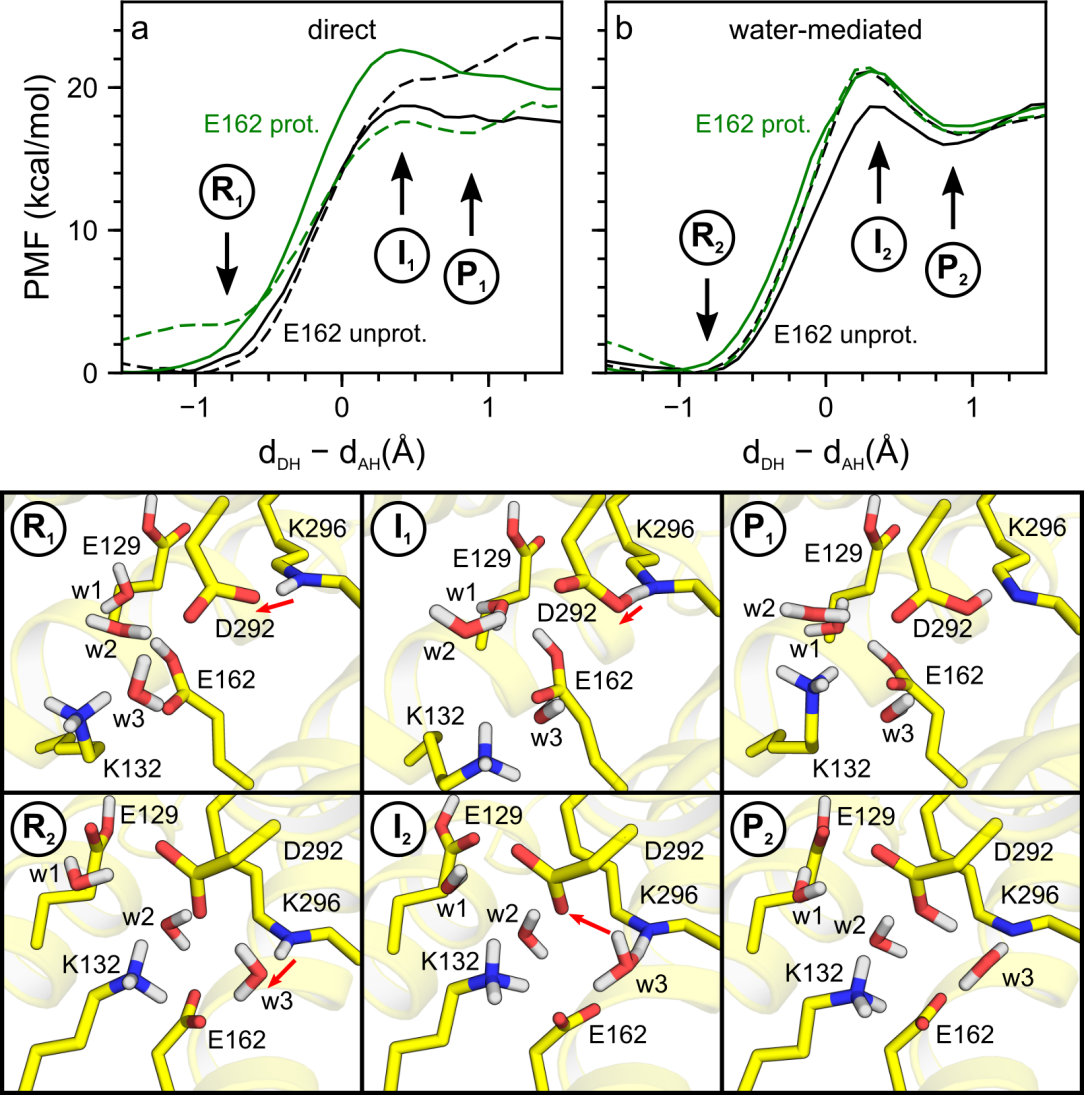
Proton transfer in wild-type C1C2. We computed PMF profiles for direct (panel a, Paths 1–4) and water-mediated (panel b, Paths 5–8) proton transfer pathways with E162 protonated (green curves) and unprotonated (black curves). PMF profiles computed for monomer 1 and monomer 2 are plotted as solid and dashed lines, respectively. Illustrations R_1_, I_1_ and P_1_ are reactant, intermediate and product states of the direct proton transfer pathway to D292, computed with E162 protonated in monomer 2 (panel a, green dashes). The red arrows in Illustrations R_1_ and I_1_ indicate the direction for proton transfer. Illustrations R_2_, I_2_ and P_2_ correspond to water-mediated proton transfer to D292 computed for monomer 2 with E162 unprotonated (black dashes in panel b).

**Fig 7:**
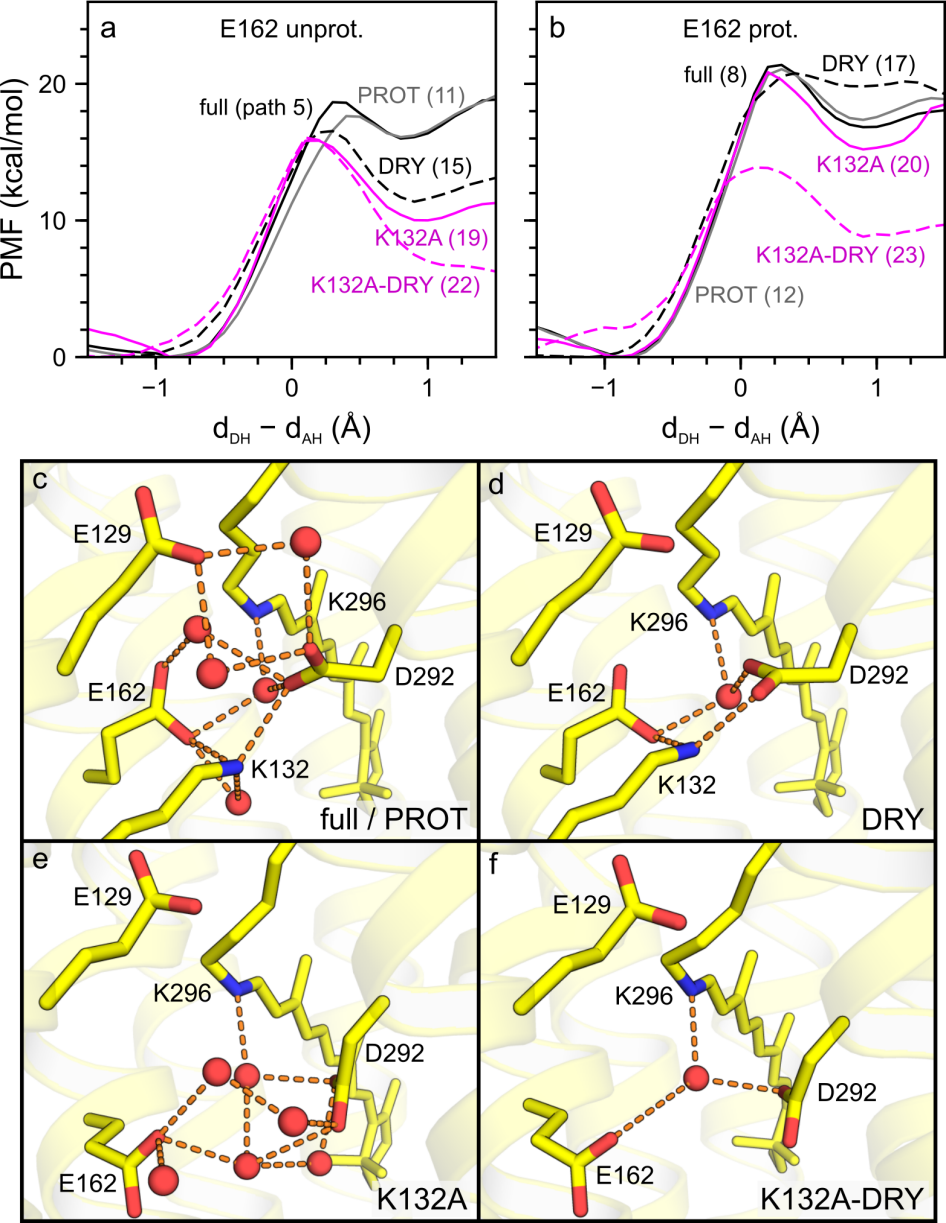
Assessing the influence of the lipid and water environment on the proton transfer energetics. We performed all computations for water-mediated proton transfer (Table 5). (a–b) PMF profiles computed for wild-type C1C2 and K132A with E162 unprotonated (a) and with E162 protonated (b). The PMF profiles were computed for the setups illustrated in panels c–f, namely: full (solid black line), PROT (grey line), DRY (black dashes), K132A (solid magenta line) and K132A-DRY (magenta dashes). (c–f) Representative snapshots for PMF calculations for: wild-type C1C2 with or without a lipid membrane (full/PROT, c); wild-type C1C2 with a lipid membrane, but with some active site waters removed (DRY, d); the K132A mutant in a lipid membrane (K132A, e); and K132A with a lipid membrane, but some active site waters removed (K132A-DRY, f). The corresponding path numbers are given in brackets (Table 5). Note that active site hydration, E162 protonation and the presence of the K132 side chain contribute to the stability of the proton on the retinal Schiff base.

**Fig 8:**
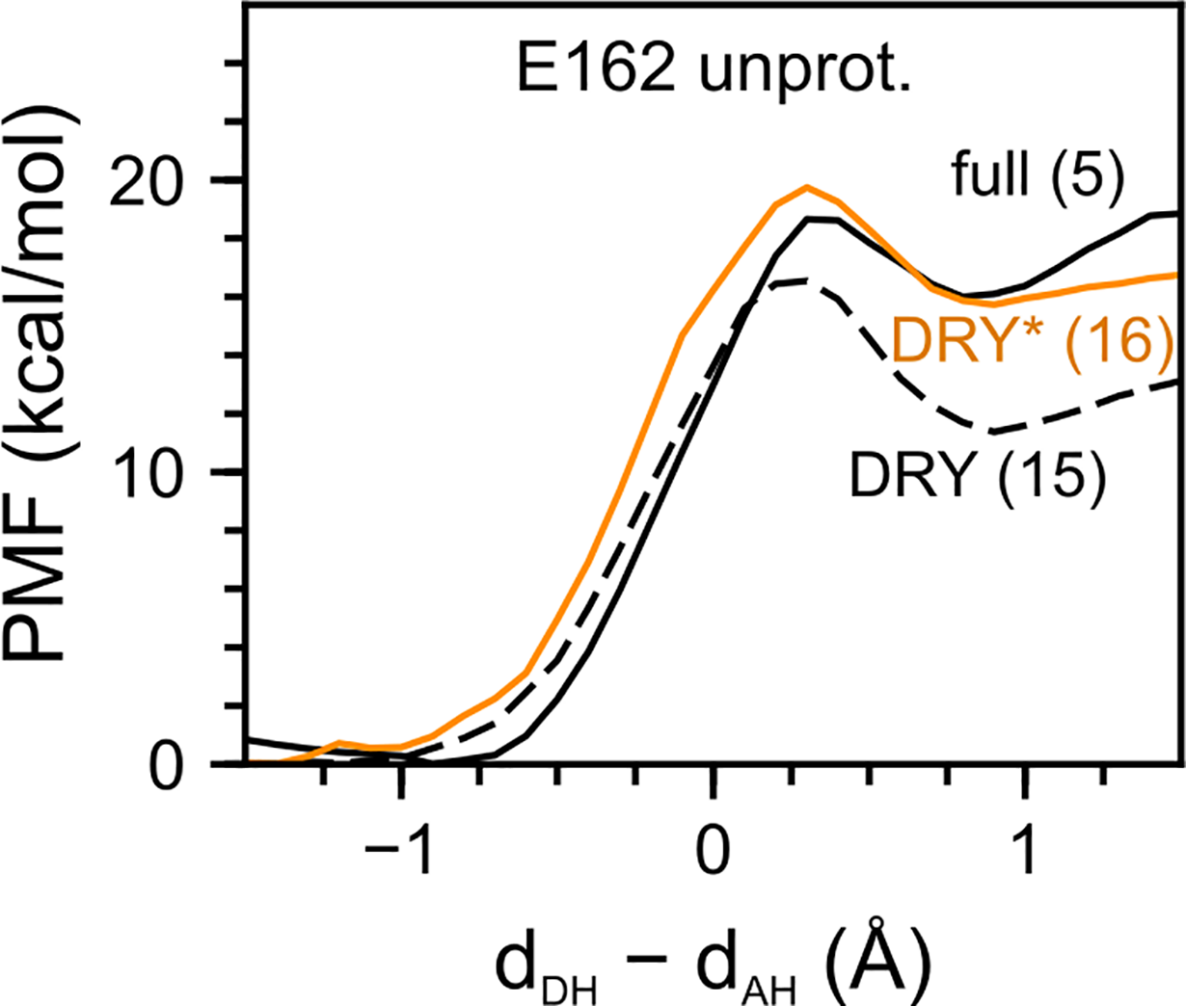
Assessing the impact of the E162 protonation on the proton transfer energetics. We computed the PMF for water-mediated proton transfer to D292 in monomer 1 of wild-type C1C2 in a hydrated lipid membrane environment (Table 5). We compared the PMF profiles of negatively charged E162 (full, solid black line, Path 5) to the PMF profile computed by first removing some of the active site water molecules (DRY, black dashes, Path 15) and the profile computed for the DRY setup, but with a proton added to E162 (DRY*, solid orange, Path 16). Note that, compared to computations with E162 unprotonated, adding a proton to E162 (DRY*) made the product state with neutral Schiff base and protonated D292 less favourable relative to the reactant state.

**Fig 9:**
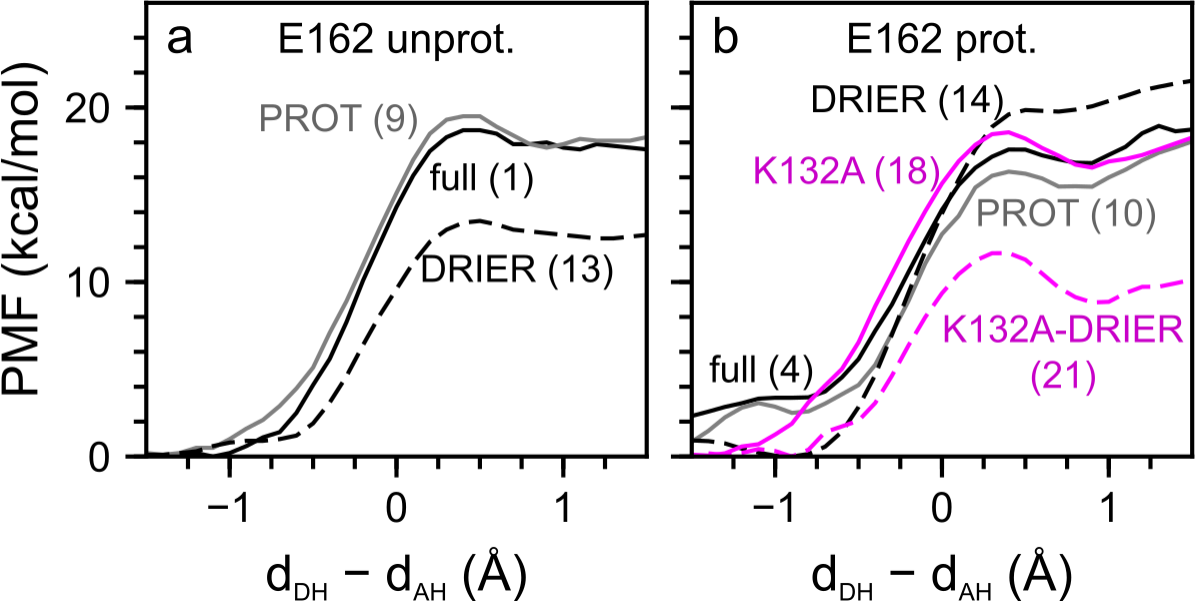
Role of water and protein electrostatic interactions in the stability of the protonated retinal Schiff base of resting state C1C2. (a) PMF profiles computed for direct proton transfer to D292 in wild-type C1C2 with unprotonated E162. We compare the PMF profiles computed for C1C2 in a hydrated lipid membrane environment (full, black line, Path 1 in Table 5), for C1C2 with lipids and bulk water removed (PROT, grey line, Path 9) and for C1C2 with all active site waters removed (DRIER, dashed line, Path 13). (b) Testing the impact of the K132A mutation on proton transfer. We show PMF profiles computed for direct proton transfer to D292, when E162 is protonated. We compare PMF profiles computed for wild-type C1C2 in the full (solid black line, Path 4), the PROT (solid grey, Path 10) and the DRIER setups (black dashes, Path 14) to PMF profiles computed for the K132A mutant in the full (solid magenta, Path 18) and the DRIER setup (magenta dashes, Path 21). We note that, for direct proton transfer in K132A with unprotonated E162, pathway calculations did not converge and were not used in the analysis.

**Fig 10:**
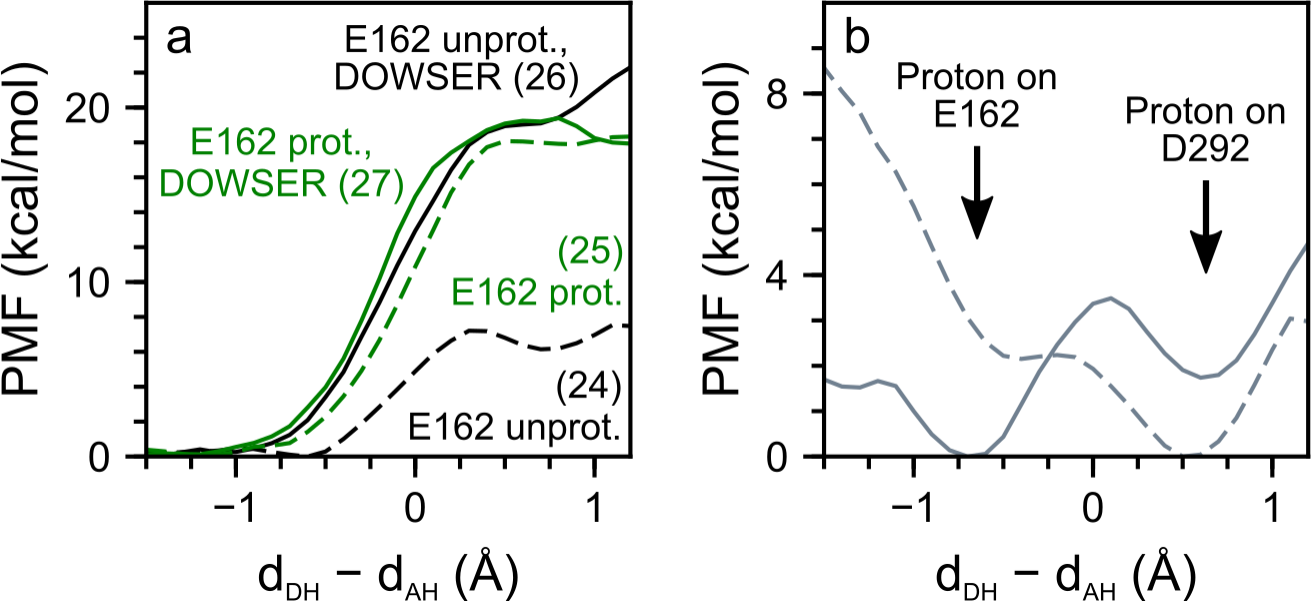
Proton transfer calculations in the crystal structure monomer of C1C2 in vacuo. (a) PMF profiles for direct transfer of the Schiff base proton to D292. In the first step, we deleted w19 (Fig 1B) und considered the remaining crystal structure water molecules for the PMF calculations with E162 unprotonated (black dashes, Path 24 in Table 5) and protonated (green dashes, Path 25). In the second step, we used DOWSER to add three water molecules to the active site of the crystal structure. We then computed PMF profiles for E162 unprotonated (solid black, Path 26) and protonated (solid green, Path 27). (b) Proton transfer from E162 to D292. We performed PMF calculations for direct proton transfer from E162 to D292 in the C1C2 crystal structure without w19 (dashes) and in the crystal structure with DOWSER-added waters (solid line). In both of these PMF computations, the Schiff base was considered protonated. Note that adding waters makes the proton prefer staying on E162 instead of D292.

The equilibrated QM/MM simulations provided the starting point for the PMF computations of proton transfer. For simplicity, we denote as reactant state (R) structures in which the proton is located on the retinal Schiff base and as product state (P) structures in which the retinal Schiff base is deprotonated and E162 and/or D292 are protonated; structures at the energy barrier of the pathways are labelled as intermediates (I, Fig 6). The proton transfer computations performed are illustrated in Figs 6–10 and are summarized in Table 5. Given the large number of paths computed here (27 in total, see Table 5), we assigned a unique path number to each PMF proton transfer calculation. In all of our setups, proton transfer calculations via water (Pathway Type 2, Fig 5) resulted in the proton being transferred to D292 (Table 5).

**Table 5:** List of proton transfer pathways with their respective reaction energies and barrier heights.

Path	Proton Transfer	Final Acceptor	Monomer	E162 Protonated	Reaction Energy	Barrier Height
*Wild type with membrane*, *active site waters present (full setup)*						
1	direct	E162	1	−	17.6	18.7
2	direct	D292	2	−	20.6	20.6
3	direct	D292	1	+	19.6	22.6
4	direct	D292	2	+	16.8	17.6
5	via water	D292	1	−	16.0	18.7
6	via water	D292	2	−	16.7	21.1
7	via water	D292	1	+	17.3	21.1
8	via water	D292	2	+	16.8	21.4
*Wild type without membrane*, *active site waters present (PROT setup)*						
9	direct	E162	1	−	17.7	19.5
10	direct	D292	2	+	15.5	16.3
11	via water	D292	1	−	16.1	17.6
12	via water	D292	2	+	17.4	21.1
*Wild type with membrane*, *all active site waters removed (DRIER setup)*						
13	direct	E162	1	−	12.5	13.5
14	direct	D292	2	+	19.8	19.9
*Wild type with membrane*, *single active site water kept (DRY setup)*						
15	via water	D292	1	−	11.4	16.5
16	via water	D292	2	+	15.7	19.7
17	via water	D292	1	+	19.8	20.8
*K132A with membrane*, *active site waters present (K132A setup)*						
18	direct	D292	2	+	16.6	18.6
19	via water	D292	1	−	10.0	15.8
20	via water	D292	2	+	15.2	20.9
*K132A with membrane*, *all active site waters removed (K132A-DRIER setup)*						
21	direct	D292	2	+	8.8	11.7
*K132A with membrane*, *single active site water kept (K132A-DRY setup)*						
22	via water	D292	1	−	6.6	16.1
23	via water	D292	2	+	8.8	13.9
*Wild type monomer in gas-phase*, *no active site waters (crystal setup)*						
24	direct	D292	—	−	6.1	7.2
25	direct	D292	—	+	17.9	18.1
*Wild type monomer in gas-phase*, *DOWSER-added waters (DOWSER setup)*						
26	direct	D292	—	−	19.0	19.0
27	direct	D292	—	+	17.9	19.4

Reaction energies and barrier heights are given in kcal/mol and have been computed for C1C2 containing all-*trans* retinal.

We found that proton transfer in wild-type C1C2, either direct or concerted via one water molecule, was associated with significant energy barriers of ∼18–23 kcal/mol and with unfavourable reaction energies of ~16–21 kcal/mol (Table 5, Fig 6); that is, the energetics of the PMF proton transfer pathways computed here was consistent with the notion that the protonated state of the retinal Schiff base is stable in all-*trans* C1C2.

The PMF computations for the direct proton transfers tend to largely lack clear valleys for the free energy minimum of the product state (Table 5, Fig 6A). This could be interpreted to suggest that, in these direct paths, the active site of C1C2 might be driven into energetically unfavourable geometries, and that longer sampling might be necessary to allow groups from the active site to adjust to the altered protonation state. By contrast, all water-mediated proton transfer pathways (Table 5, Fig 6B), display similar values of their reaction energies, and clear minima for the product state. This suggests that our PMF computations for proton transfer via a mediating water molecule were overall well converged.

A surprising observation from the PMF computations we performed was that, in simulation setups that include the hydrated lipid membrane environment of C1C2 (Fig 1A), the protonation state of E162 appeared to have had only a minor impact on the barrier for proton transfer. The water-mediated proton transfer pathways Path 6 (E162 unprotonated) and Path 7 (E162 protonated) were associated with largely the same reaction energetics (Table 5). Likewise, the direct proton transfer pathways Paths 2 and 3 had largely similar energy barriers of 20.6–22.6 kcal/mol (Table 5).

### Impact of the lipid and water environment on proton transfer energetics

To derive further insight into the structural elements essential for the stability of the protonated state of the all-*trans* retinal Schiff base, we pursued PMF computations in which we probed the role of the lipid and water environment. For these tests we used monomer 1 of the wild-type C1C2 setup with unprotonated E162 (simWu in Table 1) and monomer 2 of the setup with protonated E162 (simWp in Table 1). For either protonation state of E162, we then computed PMF profiles for: (i) the PROT setup, in which we deleted the lipid membrane and considered only the protein and water molecules within 5 Å of the protein (Fig 7A); (ii) the DRY setup, where we removed waters within 4 Å of the retinal Schiff base region, such that only a single water molecule close to the Schiff base remained (DRY, Fig 7B); and (iii) the DRY setup applied to K132A (K132A-DRY, Fig 7D). We compare these PMF profiles with the computations considering the full hydrated lipid membrane system for wild-type C1C2 (full, Fig 7A) and the K132A mutant (K132A, Fig 7C)

Comparison of the energetics of pathways initiated from reactant states that were the same except for the presence or absence of the lipid environment suggests that the lipid membrane has only a minor influence on the energetics of proton transfer from the Schiff base to the nearby carboxylate groups: Contrasting Path 1 with 9, Path 4 with 10, Path 5 with 11 and Path 8 with 12, we see that the effect that removing the lipid membrane environment has on the corresponding path energetics is within 1 kcal/mol (Table 5, Fig 7F). Such a small impact of lipids on the proton transfer energetics could be due to the fact that the proton transfer site is located close to the centre of the hydrophobic core of the lipid membrane (Fig 1A).

The PMF computations for water-mediated proton transfer in the absence of active site water other than the water closest to the Schiff base (Fig 7B) indicated that removal of waters from the active site leads to significantly lower reaction energies and energy barriers, when both E162 and D292 are negatively charged. In the case of Path 5 compared to Path 15, the energy barrier and reaction energy were lowered by 2.2 kcal/mol and 4.6 kcal/mol, respectively (Table 5, Fig 7E). The effect of removing waters from the active site is less pronounced when E162 is protonated, where the energy barrier decreased by 0.6 kcal/mol and the reaction energy increased by 3 kcal/mol (see Path 8 vs 17 in Table 5 and Fig 7F).

The computations above, on which water-mediated proton transfer was computed in the presence of a single active site water (Fig 7B), indicated that the energetics of Schiff base proton transfer is influenced significantly by waters hydrogen-bonding to the counterions. To further dissect the role of waters in Schiff base proton transfer, we performed PMF computations for direct proton transfer in which we first removed all waters within 4 Å of the Schiff base, E162 and D292, i.e. we also removed the water molecule that can hydrogen-bond to the retinal Schiff base. Comparison of Paths 1 and 13 indicated that, relative to the full setup (Path 1), the reaction energy and energy barrier for direct proton transfer to the negatively charged E162 were ~5 kcal/mol lower, when active site waters were removed (Table 5, Fig 9A). For protonated E162, removing all active site water had a smaller impact on the proton transfer energetics: Comparing Path 4 and Path 14, we see that the reaction energy and the energy barrier increased by 3.0 kcal/mol and 2.3 kcal/mol, respectively.

To further test the impact of E162 protonation, we started from the DRY setup with unprotonated E162 for water-mediated proton transfer (Path 15) and changed the protonation state of E162 by placing on its carboxylate group a proton, which had been added to the system (Path 16). We then recalculated the PMF profile using this new setup (DRY*). Protonating E162 completely negated the effect of the dehydration and increased the barrier height and reaction energy to 19.7 kcal/mol and 15.7 kcal/mol, respectively (Table 5, Fig 8). The results of this protonation test were added evidence for the strong stabilizing effect protonation of E162 has on the Schiff base proton.

### K132 as an important determinant of proton transfer energetics

The PMF results summarized above indicated that the protein and water environment largely stabilize the protonated state of the all-*trans* retinal Schiff base, such that proton transfer is energetically prohibitive. The analysis of the MM simulations (Fig 3 and S1–S3 Tables) indicated that K132 has complex hydrogen-bond dynamics, where it always hydrogen-bonds with water and where it further samples hydrogen bonds with E136, E162 and D292. We reasoned that these interactions could affect the energetics of proton transfer to E162 or D292, and consequently, studied the dynamics of the K132A mutant with MM simulations.

The overall structure of the K132A mutant, as indicated by the C_α_ RMSD profile, is largely stable (S1 Fig), i.e. at least on the timescale of our simulations, the mutation does not appear associated with large structural rearrangements of the protein. Important details of the active site geometry were, however, altered by the mutation. When E162 was negatively charged it favoured interactions with R159, and R159 could additionally hydrogen-bond to D292, E136 or H288 (S1, S2 and S4 Tables). In wild-type C1C2, R159 mainly interacted with water (S4 Table). In K132A simulations with E162 protonated, E162 hydrogen-bonded to water and N297, whereas the occupancies of hydrogen bonds to D292 and R159 were small.

As discussed above for wild-type C1C2, we used the equilibrated MM simulations of K132A to perform QM/MM MD simulations followed by PMF computations for proton transfer. We found that the mutation lowered significantly the energetic penalty for water-mediated proton transfer when E162 was negatively charged. Path 19, computed for the K132A mutant, had an energy barrier and a reaction energy smaller by 2.9 kcal/mol and 6.0 kcal/mol, respectively, than the corresponding Path 5 computed for wild-type C1C2 (Fig 7E). Removing the water molecules from inside the Schiff base region of the K132A setup (K132A-DRY, Fig 7D) led to an additional decrease in reaction energy by 3.4 kcal/mol to 6.6 kcal/mol (see Path 22 in Table 5, Fig 7E, compare to Path 5 for wild-type C1C2).

When E162 is protonated, the effect of the K132A mutation on the energetics of water-mediated proton transfer to D292 was mild—within ~1 kcal/mol (compare Paths 8 and 20 in Table 5, see also Fig 7F). This result is compatible with the PMF computation for direct proton transfer in K132A with protonated E162, for which we note that the energy barrier increased by 1.0 kcal/mol and the reaction energy remained the same as computed for wild-type C1C2 (compare Paths 4 and 18 in Table 5, see Fig 9B).

Removing waters from the active site of K132A can alter drastically the energetics of proton transfer. The reaction energy and energy barrier for water-mediated proton transfer computed for the K132A mutant with the DRY setup and protonated E162 were 8.8 kcal/mol and 13.9 kcal/mol, respectively (Path 23 in Table 5, Fig 7E and 7F); these energy values were ~6–7 kcal/mol lower than for the corresponding Path 20. Similarly, the direct proton transfer to D292 computed with protonated E162 and all active site waters removed (Path 21, K132A-DRIER) had its reaction energy and energy barrier ~7–8 kcal/mol lower than the corresponding Path 18 for K132A with full internal hydration.

Based on the computations discussed above, we conclude that K132 and active site waters help stabilize the protonated state of the all-*trans* retinal Schiff base. The stabilizing effect of the positively charged K132 side chain depends on the protonation state of E162. For unprotonated E162, the absence of K132 and a dehydrated Schiff base region were associated with the energetic penalty for proton transfer decreasing by ~10 kcal/mol. For protonated E162, removal of active site waters or the K132A mutation had milder effects on the proton transfer energetics, but, in the absence of both active site waters and of the K132 side chain, the reaction energy decreased by 8.0 kcal/mol. Consequently, to effectively stabilize the Schiff base proton, a protonated E162 seems to need at least either the K132 side chain or active site water molecules to bridge the interaction between E162 and D292.

### Proton transfer in the crystal structure

The computations presented here support an important role of water molecules as determinants of the proton transfer energetics. As an independent test of the effect of water on the proton transfer energetics, we performed an additional set of PMF computations for an isolated monomer of C1C2 starting from the crystal structure (PDB ID: 3UG9 [22]). These tests were performed for direct proton transfer using D292 as acceptor, and we considered protonated and unprotonated E162. For either protonation state of E162, we performed computations in which we used two different setups for the internal water molecules: In the first one (crystal), we removed the single crystal water that was located close to the Schiff base region (w19 in Fig 1B); in the second one (DOWSER), we considered all crystal structure waters and used DOWSER [90, 91] to add three water molecules close to the active site. Results of these test PMF computations are summarized in Table 5 (Paths 24–27).

In the crystal system with unprotonated E162, the barrier height and reaction energy were 7.2 kcal/mol and 6.1 kcal/mol, respectively (Path 24 in Table 5, Fig 10A). For protonated E162, the energy barrier was 18.1 kcal/mol and is followed by a shallow minimum with a reaction energy of 17.9 kcal/mol (Path 25 in Table 5, Fig 10A). The DOWSER setups gave similar results for both unprotonated and protonated E162 and had energy barriers of ∼19 kcal/mol and reaction energies of 19.0 kcal/mol and 17.9 kcal/mol for unprotonated and protonated E162, respectively (Path 26 and 27 in Table 5, Fig 10A).

As an additional test for the crystal structure with protonated E162, we performed a PMF computation for proton transfer from E162 to D292. For the crystal system, we found that proton transfer was favourable, with an energy barrier of 0.1 kcal/mol and a reaction energy of −2.2 kcal/mol; that is, a protonated D292 was more favourable than a protonated E162 (Fig 10B). Once water molecules were added with DOWSER, however, proton transfer from E162 to D292 had a barrier height of 3.5 kcal/mol and a reaction energy of 1.7 kcal/mol (Fig 10B), that is, the crystal structure with added water molecules favours a protonated E162 over D292. These test computations further support our proposal that water molecules can largely shape the energetics of proton transfer in the active site of C1C2.

Overall, the results of the crystal computations were compatible with the results on the effect of water molecules on the proton transfer energetics computed for the wild type and K132A in hydrated lipid membrane environments: Active site water molecules have a pronounced impact on the energetics of proton transfers computed for negatively charged E162 and only a mild effect on paths computed for neutral E162.

## Conclusions

The reaction cycles of retinal proteins involve proton transfer reactions that couple to the dynamics of the protein environment and internal water molecules. A fundamental aspect common to these proteins is the need to control the protonation state of the Schiff base of their retinal chromophore [35]. An overall productive reaction cycle requires proper timing of the proton transfer reactions and protein conformational changes that might be required, for example, to ensure proper accessibility of water to the interior of the protein. Computations with isolated model compounds indicated that the proton affinities of retinal Schiff base and acetate models strongly favour the deprotonated retinal state [54, 55]. The structure of the retinal binding pocket of ChRs, with its direct hydrogen bonds between the protonated retinal Schiff base and the carboxylate counterion(s) [22, 24] (Fig 1B), raises the important question of how the protein environment ensures stability of the protonated retinal Schiff base state. We addressed this question by performing systematic computations of proton transfer pathways using a QM/MM description of C1C2 in a hydrated lipid membrane environment. We performed independent computations with unprotonated and protonated E162, because the protonation state of the active site glutamate in ChRs has been controversial [17, 19, 22, 30].

Proton transfer from the all-*trans* retinal Schiff base to a nearby carboxylate group may occur via a direct jump of the proton or via a water molecule [29]. The energetics of the proton transfer pathways is influenced significantly by the relative orientation of the proton donor and acceptor groups and their water interactions [27, 28, 36, 55, 95, 97], as well as by protein flexibility [40] and by the protein electrostatic environment [54, 98, 99].

We accounted for the dynamics of the protein and water interactions by performing an exhaustive set of 27 PMF computations on C1C2 at room temperature, in which we considered both the direct and water-mediated proton transfers (Table 5). The ensemble of these computations indicates that the protonated state of the retinal Schiff base is energetically favourable in all-*trans* C1C2 and that transfer of the proton from the retinal Schiff base to E162 or D292 is associated with a significant energy penalty of ~16 kcal/mol in the lowest-energy water-mediated pathway (Table 5, Fig 6).

The high energetic cost of proton transfer from the retinal Schiff base to D292 in wild-type C1C2 ensures that the protein avoids unproductive deprotonation of the Schiff base prior to photoisomerization. Since the energetics of proton transfer computed without a protein environment strongly favours the transfer of the proton from the retinal Schiff base to a nearby carboxylate side chain [54, 100], the important question that arises is that of the molecular interactions that stabilize the protonated retinal Schiff base state in all-*trans* C1C2. We addressed this question by calculating proton transfer pathways for the wild type and for the K132A mutant using different amounts of active site water molecules, different protonation states of E162 and performing our computations with and without a lipid membrane.

We found that, for negatively charged E162, hydration of the Schiff base region contributes largely to the stability of the protonated Schiff base state. Removing water molecules from the vicinity of the retinal active site lowered the reaction energy for water-mediated proton transfer by ~5 kcal/mol (compare Paths 5 and 15 in Table 5). In the crystal structure of C1C2 [22], which resolves only one water molecule close to the counterion carboxylates (Fig 1B), we find that adding water increases the energetics for Schiff base deprotonation by ~13 kcal/mol (Paths 24 and 26, Table 5).

The observation that active site water interactions are essential for the stability of the protonated retinal Schiff base in C1C2 is compatible with previous work on retinal proteins [25, 27, 55]; while the observation that the active site of C1C2 contains more water molecules than indicated by the crystal structure [22] is compatible with previous experiments [19] and computations [68, 101]. Water molecules that we found to be important for the energetics of proton transfer had entered the active site region during the simulations (Fig 2). This fact highlights the importance of sampling the dynamics of membrane proteins in fluid, hydrated lipid environments.

The impact of waters on the energetics of proton transfer from the retinal Schiff base to D292 appears to depend on the protonation state of E162. In the crystal structure with protonated E162, proton transfer to D292 is 12 kcal/mol less favourable than for negatively charged E162, and the energetics remain largely the same, when water is added (Paths 25–27 in Table 5). Likewise, in membrane-embedded C1C2 with protonated E162, removing waters from the vicinity of the active site shifts the reaction energy by only ~3 kcal/mol (Paths 8 and 17 in Table 5). Changing the protonation state of E162 alters the intermolecular interactions at the active site, which in return might explain the impact of the E162 protonation state on the extent to which waters can influence the energetics of proton transfer (Figs 7, 9 and 10).

Our simulations showed that K132 has a strong effect on the proton transfer energetics in C1C2. For negatively charged E162 in K132A, the reaction energy for water-mediated deprotonation of the retinal Schiff base to D292 is 10.0 kcal/mol, which is ~6 kcal/mol less than in wild-type C1C2 (Paths 19 and 5, respectively, in Table 5). The significant role of K132 on the stability of the retinal Schiff base appears associated with its interactions with E162. In contrast to the significantly changed proton transfer energetics in K132A with negatively charged E162 (Fig 7), in the presence of protonated E162, the effect of the mutation is within 2 kcal/mol (Paths 8 and 20 in Table 5). Taken together with the significant conservation of K132 in sequences of ChRs [33] and experimental data indicating a role of K132 in controlling the charge of the counterion groups [31], the PMF computations presented here suggest that K132 is an essential determinant of the energetics of proton transfer in ChRs.

The approach used here, whereby we sampled the conformational dynamics of C1C2 and performed PMF computations on membrane-embedded C1C2 at room temperature, allows us to account explicitly for motions of the protein and its environment. In the future, we envision that such a setup could be used to perform proton transfer calculations at the proton uptake and release sites of ChR, and thus study proton transfer at the interface between proteins and lipid membranes. The atomistic description of the fluid lipid membrane accomplished in these proton transfer computations could be further used to compute the energetics of chemical reactions for enzymes embedded in membranes of different lipid compositions, and thus decipher the role that specific lipid molecules might have in the reaction coordinates. In the case of ChRs, simulations with better models of the cell membrane might be important for studying the energetics of proton uptake and release, which involve groups closer to the interface of the lipid membrane.

## Supporting information

S1 Fig RMSD of the backbone C_α_ atoms of C1C2. (a) RMSD profile for wild-type C1C2 with unprotonated E162 (simWu). The RMSD computed from the last 50 ns of simWu is 2.6 ± 0.1 Å for the full protein (dark green), as compared to 1.1 ± 0.1 Å and 3.7 ± 0.2 Å for the α-helical regions (dark purple) and loops (dark slate grey), respectively. (b) RMSD profile for the K132A mutant simulation with unprotonated E162 (simMu). (c) RMSD profile for wild-type C1C2 with protonated E162 (simWp). (d) RMSD profile for K132 with protonated E162 (simMp). Results from the corresponding repeat simulations are shown in brighter colours.
(DOCX)

S2 Fig Water inside the Schiff base region in simulations of wild-type C1C2 with unprotonated (simWu) and protonated E162 (simWp). The number of water molecules hydrogen-bonding to the Schiff base or the side chains of E162 or D292 is shown in blue and the distance between NZ of K132 and CG of D292 is shown in red. (a, d) Data from the repeat simulation of wild-type C1C2 with unprotonated E162 for monomer 1 (a) and monomer 2 (d). (b, e) Data from the simulation with protonated E162 (simWp). (c, f) Data from the repeat simulation of wild-type C1C2 with protonated E162 (simWp′).
(DOCX)

S3 Fig Violin plots of the distance between CD of E162 and CG of D292. Violin plots have been calculated for the last 50 ns of each simulation, with simulations labels according to Table 1. The inlayed box-and-whiskers plots indicate the 5^th^, 25^th^, 50^th^, 75^th^ and 95^th^ percentile.
(DOCX)

S4 Fig Interactions between K132 and the counterions E162/D292. (a) K132 hydrogen-bonding to both E162 and D292. (b) K132 hydrogen-bonding to a single counterion (E162) only.
(DOCX)

S1 Table Hydrogen bonding partners of the E162 carboxyl(ate). Percentages have been computed for the last 50 ns of the MM simulations. For clarity, only percentages >3% are shown. A prime symbol indicates repeat simulations.
(DOCX)

S2 Table Hydrogen bonding partners of the D292 carboxyl(ate). Percentages have been computed for the last 50 ns of the MM simulations. For clarity, only percentages >3% are shown. A prime symbol indicates repeat simulations.
(DOCX)

S3 Table Hydrogen bonding partners of the K132 ammonium group. Percentages have been computed for the last 50 ns of the MM simulations. For clarity, only percentages >3% are shown. A prime symbol indicates repeat simulations.
(DOCX)

S4 Table Hydrogen bonding partners of the R159 side chain amine and imine groups. Percentages have been computed for the last 50 ns of the MM simulations. For clarity, only percentages >3% are shown. A prime symbol indicates repeat simulations.
(DOCX)
