# Supplementary material for: Mechanism by which water and protein electrostatic interactions control proton transfer at the active site of channelrhodopsin
Source: PLoS One. 2018 Aug 7;13(8):e0201298. doi: 10.1371/journal.pone.0201298 (PMC6080761; doi:10.1371/journal.pone.0201298)
Supplement: S1 Fig — (a) RMSD profile for wild-type C1C2 with unprotonated E162 (simWu). The RMSD computed from the last 50 ns of simWu is 2.6 ± 0.1 Å for the full protein (dark green), as compared to 1.1 ± 0.1 Å and 3.7 ± 0.2 Å for the α-helical regions (dark purple) and loops (dark slate grey), respectively. (b) RMSD profile for the K132A mutant simulation with unprotonated E162 (simMu). (c) RMSD profile for wild-type C1C2 with protonated E162 (simWp). (d) RMSD profile for K132 with protonated E162 (simMp). Results from the corresponding repeat simulations are shown in brighter colours. (DOCX) [file pone.0201298.s001.docx]

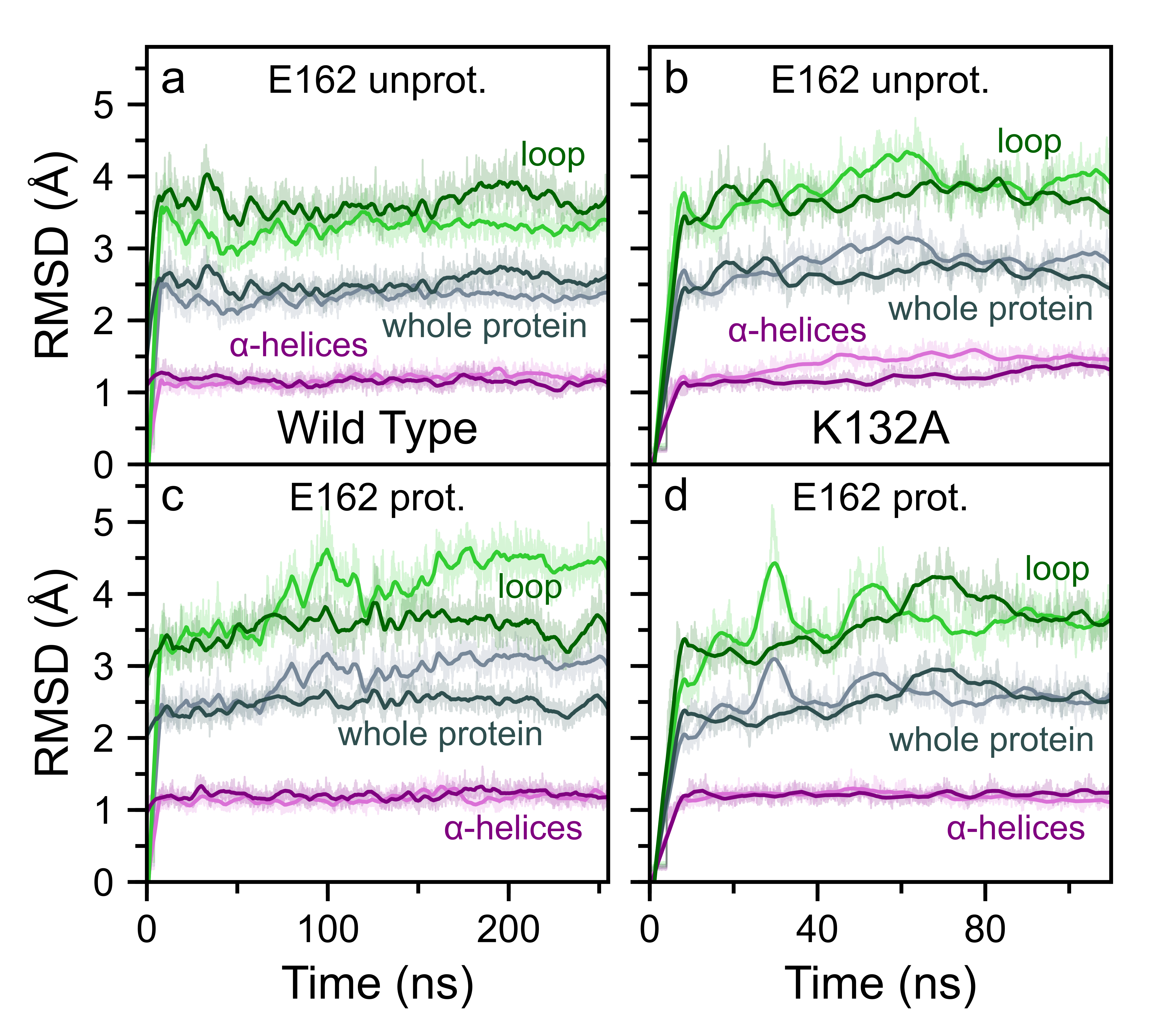


**S1 Fig. RMSD of the Backbone C_α_ Atoms of C1C2.** (a) RMSD profile for wild-type C1C2 with unprotonated E162 (simWu). The RMSD computed from the last 50 ns of simWu is 2.6 ± 0.1 Å for the full protein (dark green), as compared to 1.1 ± 0.1 Å and 3.7 ± 0.2 Å for the α-helical regions (dark purple) and loops (dark slate grey), respectively. (b) RMSD profile for the K132A mutant simulation with unprotonated E162 (simMu). (c) RMSD profile for wild-type C1C2 with protonated E162 (simWp). (d) RMSD profile for K132 with protonated E162 (simMp). Results from the corresponding repeat simulations are shown in brighter colours.
