# Supplementary material for: Mechanism by which water and protein electrostatic interactions control proton transfer at the active site of channelrhodopsin
Source: PLoS One. 2018 Aug 7;13(8):e0201298. doi: 10.1371/journal.pone.0201298 (PMC6080761; doi:10.1371/journal.pone.0201298)
Supplement: S2 Fig — The number of water molecules hydrogen-bonding to the Schiff base or the side chains of E162 or D292 is shown in blue and the distance between NZ of K132 and CG of D292 is shown in red. (a, d) Data from the repeat simulation of wild-type C1C2 with unprotonated E162 for monomer 1 (a) and monomer 2 (d). (b, e) Data from the simulation with protonated E162 (simWp). (c, f) Data from the repeat simulation of wild-type C1C2 with protonated E162 (simWp′). (DOCX) [file pone.0201298.s002.docx]

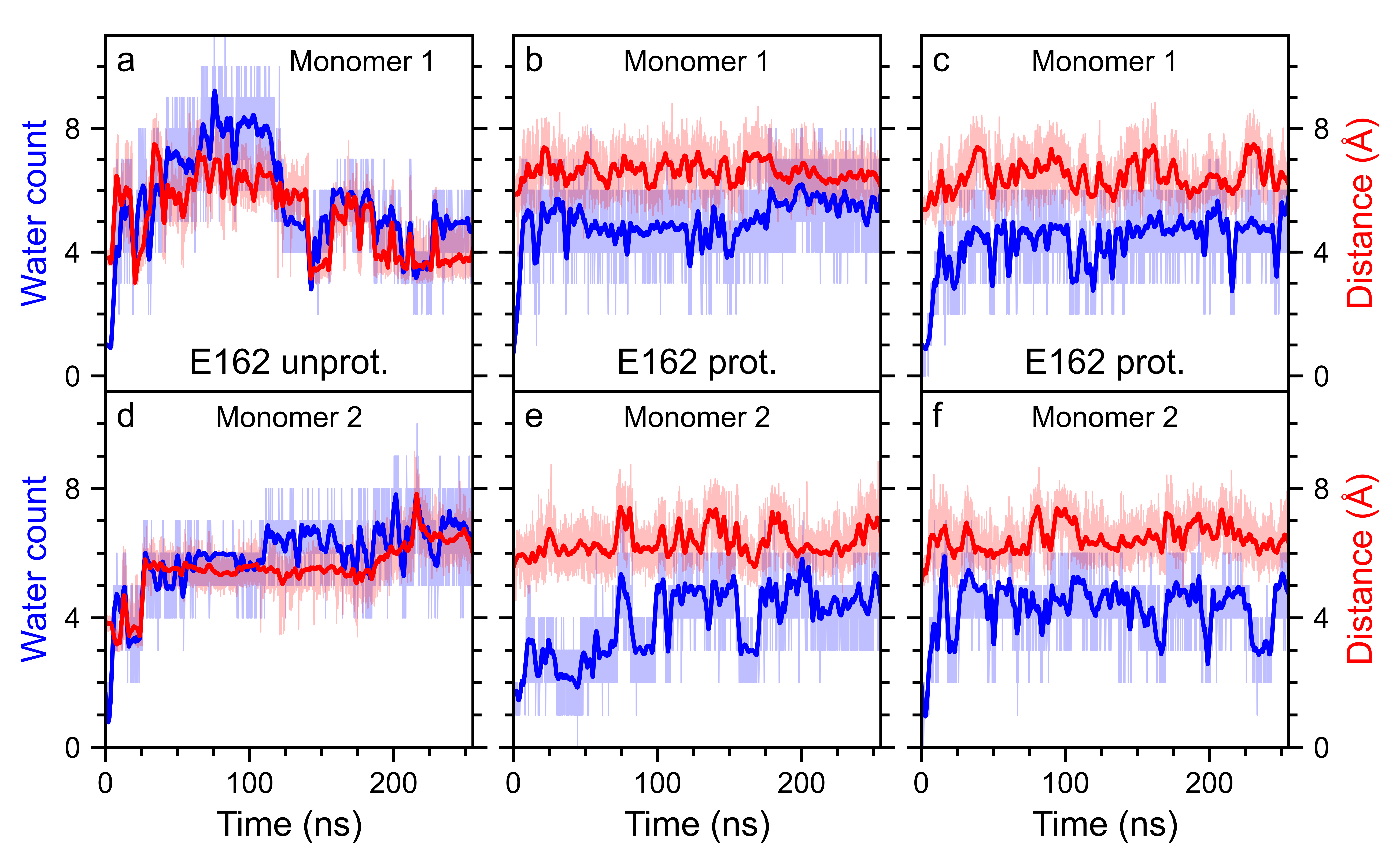


**S2 Fig. Water inside the Schiff Base Region in Simulations of Wild-Type C1C2 with Unprotonated (simWu) and Protonated E162 (simWp).** The number of water molecules hydrogen-bonding to the Schiff base or the side chains of E162 or D292 is shown in blue and the distance between NZ of K132 and CG of D292 is shown in red. (a, d) Data from the repeat simulation of wild-type C1C2 with unprotonated E162 for monomer 1 (a) and monomer 2 (d). (b, e) Data from the simulation with protonated E162 (simWp). (c, f) Data from the repeat simulation of wild-type C1C2 with protonated E162 (simWp′).
