# Supplementary material for: Mechanism by which water and protein electrostatic interactions control proton transfer at the active site of channelrhodopsin
Source: PLoS One. 2018 Aug 7;13(8):e0201298. doi: 10.1371/journal.pone.0201298 (PMC6080761; doi:10.1371/journal.pone.0201298)
Supplement: S3 Fig — Violin plots have been calculated for the last 50 ns of each simulation, with simulations labels according to Table 1. The inlayed box-and-whiskers plots indicate the 5th, 25th, 50th, 75th and 95th percentile. (DOCX) [file pone.0201298.s003.docx]

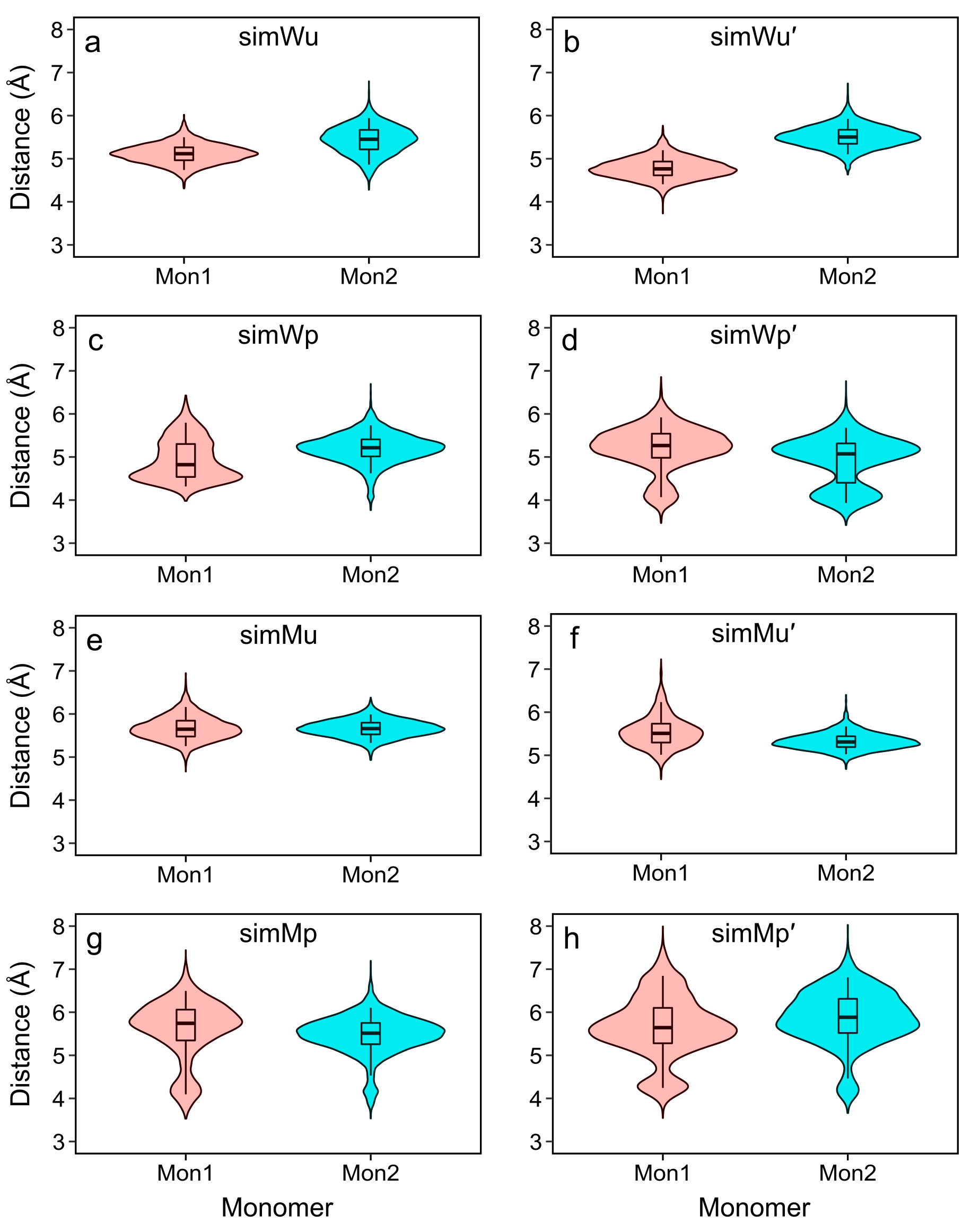


**S3 Fig. Violin Plots of the Distance between CD of E162 and CG of D292.** Violin plots have been calculated for the last 50 ns of each simulation, with simulations labels according to Table 1. The inlayed box-and-whiskers plots indicate the 5^th^, 25^th^, 50^th^, 75^th^ and 95^th^ percentile.
