# Supplementary material for: Mechanism by which water and protein electrostatic interactions control proton transfer at the active site of channelrhodopsin
Source: PLoS One. 2018 Aug 7;13(8):e0201298. doi: 10.1371/journal.pone.0201298 (PMC6080761; doi:10.1371/journal.pone.0201298)
Supplement: S4 Fig — (a) K132 hydrogen-bonding to both E162 and D292. (b) K132 hydrogen-bonding to a single counterion (E162) only. (DOCX) [file pone.0201298.s004.docx]

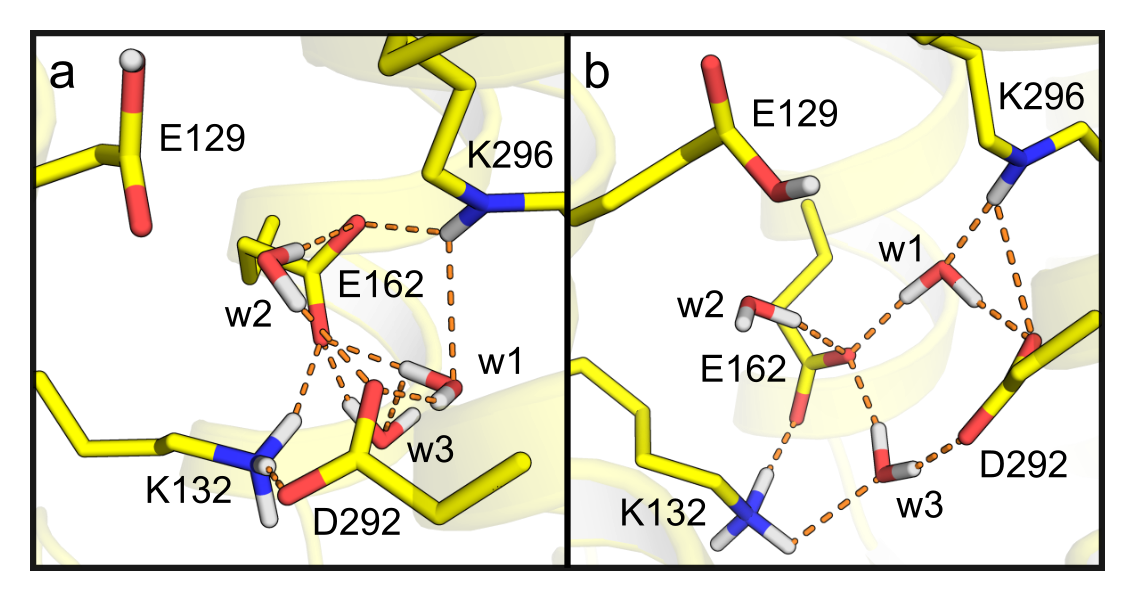


**S4 Fig. Interactions between K132 and the Counterions E162/D292.** (a) K132 hydrogen-bonding to both E162 and D292. (b) K132 hydrogen-bonding to a single counterion (E162) only.
