# Supplementary material for: Mechanism by which water and protein electrostatic interactions control proton transfer at the active site of channelrhodopsin
Source: PLoS One. 2018 Aug 7;13(8):e0201298. doi: 10.1371/journal.pone.0201298 (PMC6080761; doi:10.1371/journal.pone.0201298)
Supplement: S1 Table — Percentages have been computed for the last 50 ns of the MM simulations. For clarity, only percentages >3% are shown. A prime symbol indicates repeat simulations. (DOCX) [file pone.0201298.s005.docx]

**S1 Table. Hydrogen Bonding Partners of the E162 carboxyl(ate).**

| **Sim** | **Monomer 1 (%)** | | | | | **Monomer 2 (%)** | | | | |
| --- | --- | --- | --- | --- | --- | --- | --- | --- | --- | --- |
|  | **Water** | **K132** | **R159** | **D292** | **N297** | **Water** | **K132** | **R159** | **D292** | **N297** |
| simWu | 100 | 36 | — | — | 92 | 100 | 82 | — | — | 8 |
| simWu′ | 100 | 100 | — | — | 50 | 100 | 93 | — | — | — |
| simWp | 99 | 41 | — | 45 | 12 | 100 | 46 | — | — | 13 |
| simWp′ | 96 | 43 | — | 13 | 4 | 93 | 36 | — | 25 | 12 |
| simMu | 100 | — | 100 | — | — | 100 | — | 100 | — | 31 |
| simMu′ | 100 | — | 26 | — | 28 | 100 | — | 99 | — | — |
| simMp | 100 | — | — | 13 | — | 100 | — | — | 5 | — |
| simMp′ | 99 | — | — | 12 | — | 99 | — | — | 6 | — |

Percentages have been computed for the last 50 ns of the MM simulations. For clarity, only percentages >3 % are shown. A prime symbol indicates repeat simulations.
