# Supplementary material for: Mechanism by which water and protein electrostatic interactions control proton transfer at the active site of channelrhodopsin
Source: PLoS One. 2018 Aug 7;13(8):e0201298. doi: 10.1371/journal.pone.0201298 (PMC6080761; doi:10.1371/journal.pone.0201298)
Supplement: S3 Table — Percentages have been computed for the last 50 ns of the MM simulations. For clarity, only percentages >3% are shown. A prime symbol indicates repeat simulations. (DOCX) [file pone.0201298.s007.docx]

**S3 Table. Hydrogen Bonding Partners of the K132 Ammonium Group.**

| **Sim** | **Monomer 1 (%)** | | | | | **Monomer 2 (%)** | | | | |
| --- | --- | --- | --- | --- | --- | --- | --- | --- | --- | --- |
|  | **Water** | **K132** | **E136** | **E162** | **D292** | **Water** | **K132** | **E136** | **E162** | **D292** |
| simWu | 100 | — | 66 | 33 | 32 | 100 | — | 45 | 80 | — |
| simWu′ | 98 | — | — | 100 | 73 | 100 | 25 | 6 | 91 | — |
| simWp | 100 | — | 99 | 5 | — | 100 | — | 99 | — | — |
| simWp′ | 100 | — | 98 | — | — | 100 | — | 97 | — | — |
| simMu | — | — | — | — | — | — | — | — | — | — |
| simMu′ | — | — | — | — | — | — | — | — | — | — |
| simMp | — | — | — | — | — | — | — | — | — | — |
| simMp′ | — | — | — | — | — | — | — | — | — | — |

Percentages have been computed for the last 50 ns of the MM simulations. For clarity, only percentages >3 % are shown. A prime symbol indicates repeat simulations.
