# Supplementary material for: Mechanism by which water and protein electrostatic interactions control proton transfer at the active site of channelrhodopsin
Source: PLoS One. 2018 Aug 7;13(8):e0201298. doi: 10.1371/journal.pone.0201298 (PMC6080761; doi:10.1371/journal.pone.0201298)
Supplement: S4 Table — Percentages have been computed for the last 50 ns of the MM simulations. For clarity, only percentages >3% are shown. A prime symbol indicates repeat simulations. (DOCX) [file pone.0201298.s008.docx]

**S4 Table. Hydrogen Bonding Partners of the R159 Side Chain Amine and Imine Groups.**

| **Sim** | **Monomer 1 (%)** | | | | | | **Monomer 2 (%)** | | | | | |
| --- | --- | --- | --- | --- | --- | --- | --- | --- | --- | --- | --- | --- |
|  | **Water** | **E162** | **D292** | **K132** | **E136** | **H288** | **Water** | **E162** | **D292** | **K132** | **E136** | **H288** |
| simWu | 100 | — | — | — | 4 | — | 100 | — | — | — | — | 8 |
| simWu′ | 100 | — | — | — | — | 10 | 100 | — | — | 25 | 100 | — |
| simWp | 100 | — | — | — | — | — | 100 | — | — | — | — | — |
| simWp′ | 100 | — | — | — | — | 10 | 100 | — | — | — | — | 6 |
| simMu | 100 | 100 | — | — | 87 | — | 100 | 100 | 99 | — | — | — |
| simMu′ | 100 | 26 | 10 | — | 85 | — | 100 | 99 | 100 | — | — | 100 |
| simMp | 100 | — | — | — | 8 | 67 | 100 | — | — | — | 100 | — |
| simMp′ | 100 | — | 4 | — | — | 30 | 100 | — | — | — | — | 10 |

Percentages have been computed for the last 50 ns of the MM simulations. For clarity, only percentages >3 % are shown. A prime symbol indicates repeat simulations.
